# Ultrasensitive Detection of Neurofilament Light in Plasma Using F(Ab’)_2_‐Modified Graphene Field‐Effect Biosensor

**DOI:** 10.1002/smll.73928

**Published:** 2026-05-26

**Authors:** Selvinaz Burcu Kizilates, Rica Asrosa, Lenart Senicar, Anuja Sharma, Seda Gungordu Er, Nisha Naeem, Ahmad Nizamuddin bin Muhammad Mustafa, Yang Wu, Neil Graham, Amanda Heslegrave, Mohan Edirisinghe, Antonio Lombardo, Elias Torres, Henrik Zetterberg, Sami Ramadan, David J. Sharp, Bing Li

**Affiliations:** ^1^ Institute For Materials Discovery University College London London UK; ^2^ Department of Physics Universitas Sumatera Utara Medan Indonesia; ^3^ UK Dementia Research Institute University College London London UK; ^4^ Department of Mechanical Engineering University College London London UK; ^5^ Department of Electronic and Electrical Engineering University College London London UK; ^6^ Department of Materials Imperial College London London UK; ^7^ FTKEK Universiti Teknikal Malaysia Melaka Melaka Malaysia; ^8^ Electrical Engineering Division University of Cambridge Cambridge UK; ^9^ Department of Brain Sciences Imperial College London London UK; ^10^ Care Research and Technology Centre UK Dementia Research Institute London UK; ^11^ Institute of Neurology University College London London UK; ^12^ London Centre for Nanotechnology London UK; ^13^ Graphenea Semiconductor San Sebastián Spain; ^14^ Department of Psychiatry and Neurochemistry University of Gothenburg Mölndal Sweden; ^15^ Clinical Neurochemistry Laboratory Sahlgrenska University Hospital Mölndal Sweden; ^16^ Hong Kong Centre for Neurodegenerative Diseases Hong Kong China; ^17^ Centre For Brain Research Indian Institute of Science Bangalore India; ^18^ Department of Pathology and Laboratory Medicine School of Medicine and Public Health University of Wisconsin Madison Wisconsin USA; ^19^ Wisconsin Alzheimer's Disease Research Center University of Wisconsin School of Medicine and Public Health University of Wisconsin Madison Wisconsin USA

**Keywords:** 1‐Pyrenebutyric acid N‐hydroxysuccinimide ester, biosensors, Debye length, F(ab’)_2_ antibody fragmentation, graphene field‐effect transistors

## Abstract

Neurofilament light (NfL) is a discriminative blood biomarker for many neurological diseases. Current accurate analysis relating to NfL relies on state‐of‐the‐art technologies such as the single‐molecule array (Simoa) and immunoprecipitation‐mass spectrometry (IP‐MS), which require complicated machinery, skilled operational personnel, and well‐equipped laboratories. Herein, we demonstrate a robust on‐chip graphene field‐effect transistor (GFET) biosensing platform for the ultrasensitive detection of NfL. This work utilizes smaller antibody fragments F(ab’)_2_ to mitigate Debye screening and enhance sensing performance, alongside quantitative characterization of 1‐pyrenebutyric acid N‐hydroxysuccinimide ester (PBASE) surface density to support controlled antibody immobilization. Compared with whole antibody‐based GFETs, this F(ab’)_2_‐modified GFET platform is shown to achieve a 114% increase in sensitivity, a fivefold improvement in the limit‐of‐detection (LoD) down to 0.18 pg/mL, and a wide dynamic detection range from 0.18 to 1500 pg/mL, together with good selectivity, stability, and reproducibility. This biosensing platform is validated against Simoa technology for the detection of NfL in clinical plasma samples, yielding a high correlation coefficient of 0.99. These results demonstrate the potential of GFETs for point‐of‐care diagnosis and the monitoring of neurological diseases in frontline clinical settings, outperforming conventional immunoassays and approaching Simoa sensitivity.

## Graphical abstract

An antibody fragment (F(ab')_2_)‐modified graphene field‐effect transistor biosensor is developed for the ultrasensitive detection of neurofilament light chain in clinical plasma. By reducing the Debye screening effect, the platform achieves a 114% sensitivity increase and a fivefold lower detection limit compared to whole‐antibody approaches, benchmarked against state‐of‐the‐art single‐molecule array technology.

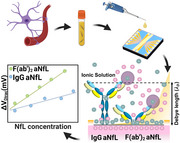

## 1 Introduction

Neurological diseases affect millions of individuals globally and are associated with substantial financial burdens upon healthcare systems in both developed and developing countries [1]. The current diagnosis and classification of neurological diseases now benefit greatly from the analysis of pathological biomarkers [2], and great progress has recently been made in the discovery of relevant novel blood biomarkers [3, 4, 5]. For example, neurofilament light (NfL) has been found to be an important biomarker for a range of neurological diseases such as traumatic brain injury (TBI) [6, 7]. Its blood concentration starts to increase before symptoms appear and fluctuates as the disease progresses, making it an ideal biomarker for early diagnosis and progression monitoring. However, state‐of‐the‐art technologies for the precise determination of these biomarkers rely on advanced immunoassays, such as the single molecule array (Simoa) [8], and immunoprecipitation‐mass spectrometry (IP‐MS) [9]. These technologies present considerable implementation challenges due to their reliance on complex fluorescent labelling protocols, laser excitation and signal acquisition instrumentation, specialised technical expertise, and high operational costs, which limit their adoption in primary care facilities. Therefore, it is of great importance to develop more reliable, cost‐effective, and accessible technologies for the point‐of‐care (POC) analysis of these important biomarkers.

Graphene field‐effect transistors (GFETs) have emerged as a promising analytical method for the detection of neurological disease biomarkers at extremely low concentrations [3, 10]. This technology operates on fully electronic principles and features a compact, stand‐alone design, which enables POC diagnostics and rapid deployment compared with conventional assay platforms. The standard GFET biosensor consists of three components: a graphene channel that converts biomolecular interactions into electrical signals [11], a linker molecule that attaches to graphene via covalent or noncovalent interactions and provides functional groups for probe attachment [12], and a probe molecule that serves as the biorecognition element for selective target binding [13]. Although this architecture serves as the foundation for most GFET research, it has several challenges that must be addressed before the technology can be used in practical applications.

One challenge is the understanding and control of the molecular density of linkers on the graphene surface, as this parameter ultimately limits probe molecule coverage. 1‐pyrenebutyric acid N‐hydroxysuccinimide ester (PBASE) is commonly used as a linker, and its pyrene moiety is adsorbed onto graphene via π–π interactions while the NHS ester interacts with primary amines on antibodies. The quantitative study of linker density is therefore critical in probe immobilization and, consequently, sensor performance. However, studies of PBASE molecular density have rarely been reported, and the systematic research required will be essential in ensuring controlled antibody immobilization and consistent biosensor performance [14]. Silvesteri et al., studied the molecular density of PBASE utilizing a quartz crystal microbalance with dissipation (QCM‐D) method involving a functionalization density of 1.62 molecules per nm^2^ [15]. The spectrophotometric study by Lozano‐Chamizo et al., quantified the adsorbed PBASE, with the highest reported value of 4.7 × 10^−11^ mol for 100 µM PBASE [16]. Similarly, Mishyn et al., evaluated the incubation time of PBASE using cyclic voltammetry and found that 2 h of incubation led to an optimal surface coverage of 6.04 ± 1.01 × 10^−11^ mol cm^−2^, whilst over 6 h resulted in a significant decrease in coverage, which was attributed to the hydrolyzation of the ester group of PBASE. Another challenge is the Debye length (*λ_D_*), which describes how the distance over which the net electrostatic effects of charge carriers persist. This can influence the electrical current passing through the graphene channel, thereby directly affecting the sensitivity and limit of detection (LoD) of a GFET biosensor, and is defined in Equation (1):

(1) $$\lambda_{D}=\sqrt{\frac{\varepsilon k_{B}T}{q^{2}c}}\;$$

where *ε* is the dielectric permittivity of the medium, *k_B_* is Boltzmann's constant, *T* is temperature, *q* is the electron charge, and *c* represents the ionic strength of the electrolyte. In physiological solutions such as blood, the ionic strength is typically around 150 mM, resulting in a value of *λ_D_* of approximately 1 nm [17], beyond which the electrostatic potential of a charged molecule is effectively screened by surrounding counter‐ions. Most probe molecules, such as whole IgG antibodies, position the binding events approximately 10–15 nm above the graphene surface, placing them far beyond the Debye length, resulting in significant electrostatic screening of the signal. Although efforts have been made to extend the Debye length by reducing the ionic strength of clinical samples, the improvement achieved is insufficient relative to the size of whole antibodies. This necessitates additional sample preparation, which is undesirable for POC diagnostics [18, 19, 20, 21]. Another strategy to tackle the short Debye length is to use smaller probe molecules such as antibody fragments Fab (∼3–5 nm) and F(ab’)_2_ (∼5 nm). These are significantly smaller than whole IgG (∼15 nm) [22], and they can potentially bring more charged biomarkers within the Debye length. Elnathan et al. demonstrated that Fab and F(ab’)_2_ fragments improved detection sensitivity to within the sub‐pM range [23], while Andoy et al. successfully detected thyroid‐stimulating hormone in whole serum using F(ab’)_2_ fragments on GFETs, with a LoD of 10 fM [24]. Likewise, Fab fragments have been successfully applied to GFETs, significantly enhancing their sensitivity [25, 26]. However, this strategy has not been applied for the ultrasensitive detection of NfL.

Here, we describe the development of a robust on‐chip GFET biosensing platform that employs F(ab’)_2_ fragments as probe molecules for the ultrasensitive detection of NfL. A thorough characterisation of PBASE molecular density was performed to understand immobilization of F(ab’)_2_ fragments on the graphene surface. F(ab’)_2_ fragments were produced from whole IgG antibodies through the enzymatic digestion of the Fc region, followed by purification. Compared with whole IgG‐based GFETs, our F(ab’)_2_‐based platform demonstrated markedly enhanced sensitivity and a lower LoD at the femtomolar level by effectively mitigating the Debye screening effect. This biosensing platform was further validated against Simoa technology using patient plasma samples, demonstrating excellent correlation and thus highlighting its potential for the POC monitoring of neurological diseases.

## 2 Results and Discussion

**FIGURE 1:**
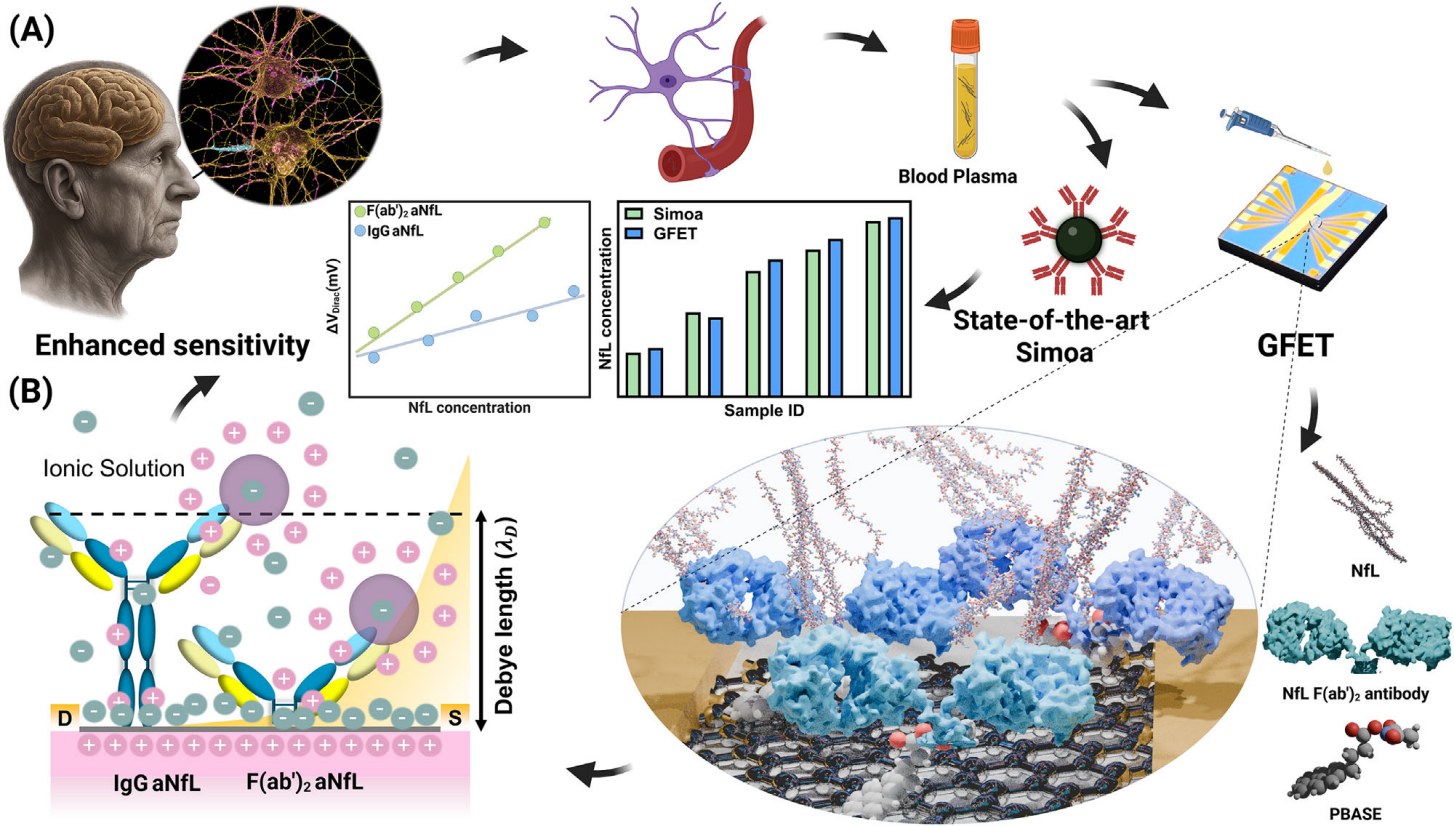
Schematic of the F(ab’)_2_‐modified GFET platform for NfL detection. (A) Levels of NfL are elevated following brain injury, and it circulates in the bloodstream at trace levels. Its detection in patient plasma was performed using the GFET platform, and the results were cross‐validated with those using the Simoa platform. (B) The zoom‐in on the right shows the graphene channel and functionalization components, and a further magnification on the left illustrates molecular‐level interactions where F(ab’)_2_ fragments were used instead of IgG in order to remain within the Debye length and enhance sensitivity.

A schematic of the GFET platform used in this work is shown in Figure 1. As illustrated in Figure 1A, NfL is a brain‐based biomarker associated with neurological diseases which circulates at low concentrations in plasma by crossing the blood‐brain barrier. The GFET enables ultrasensitive, label‐free detection and was cross‐validated against the gold‐standard Simoa platform. Figure 1B shows a magnified illustration of the graphene channel and the components employed for surface functionalization. To investigate the functional biointerface, PBASE density was quantified to support controlled antibody immobilization. A further zoom highlights the molecular‐level interactions, where the use of F(ab’)_2_ fragments rather than full‐length IgG allows binding to occur within the Debye length, thereby enhancing sensitivity by minimising Debye screening. An optical image of the GFET platform with labelled features and a scale bar is provided in Figure S1.

### 2.1 Molecular Density of PBASE

**FIGURE 2:**
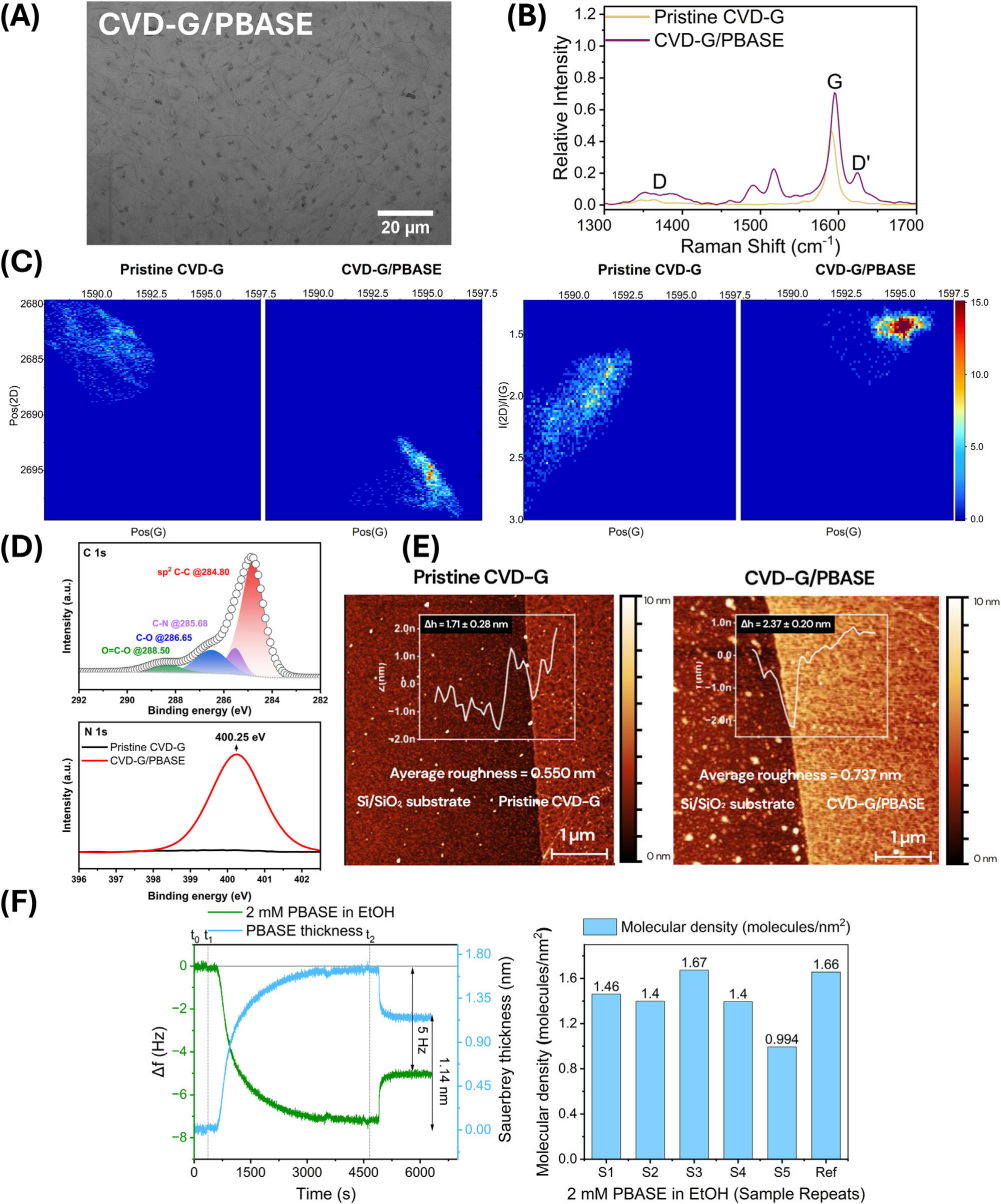
Confirmation of PBASE functionalization and quantification of PBASE molecular density on the graphene surface. (A) SEM image of PBASE‐functionalized graphene. (B) The zoomed‐in Raman spectra confirm the existence of PBASE as the linker molecule, showing the changes in comparison to pristine CVD‐G. (C) Spatial mapping of 10 × 10 µm^2^ graphene surfaces to investigate the effect of PBASE functionalization. Left: 2D histograms of Pos(2D) and Pos(G) of pristine and PBASE functionalized graphene, respectively. Right: 2D histograms of I(2D)/I(G) and Pos(G) of pristine and PBASE functionalized graphene, respectively. The distribution frequency of the values obtained is represented by a color bar from blue (0) to red (15). (D) XPS analysis of C 1s and N 1s peaks confirms the functionalization of PBASE on the graphene surface. (E) AFM was used to measure the thickness of the graphene and PBASE layer. (F) QCM‐D was employed to assess the coverage of the PBASE layer and its thickness on the graphene surface.

Multiple characterization techniques were employed to verify the successful adsorption of PBASE onto chemical vapor deposited graphene (CVD‐G), then to assess the resulting changes in surface properties, and finally to quantify PBASE molecular density, as shown in Figure 2. Scanning electron microscopy (SEM) imaging of CVD‐G after PBASE treatment showed a continuous monolayer structure (Figure 2A), with minor defects that did not disrupt continuity. Raman spectroscopy was used to confirm PBASE functionalization and to assess its impact on graphene. Pristine monolayer CVD‐G exhibited the expected G peak (at 1587 cm^−1^), the 2D peak (at 2683 cm^−1^), and a negligible D peak (∼1340 cm^−1^) [27]. Following PBASE adsorption, single‐point Raman measurements showed upshifts of 4 cm^−1^ for the G peak and 8 cm^−1^ for the 2D peak (Figure 2B), together with a weak D′ shoulder (∼1625 cm^−1^) and an increase in I_D_/I_G_ from 0.07 to 0.19. This is consistent with non‐covalent adsorption, introducing slight additional scattering. The corresponding I_D_/I_G_ and I_2D_/I_G_ ratios and the full‐range Raman spectrum of CVD‐G/PBASE are presented in Table S1 and Figure S5, respectively. The Raman mapping of pristine CVD‐G (10 × 10 µm, 2116 spectra) revealed a data cluster with Pos(G) in the range of ∼1590–1593 cm^−1^ and Pos(2D) between ∼2680–2688 cm^−1^, as indicative of lightly doped, low‐strain material. Following PBASE functionalization, the distribution shifted systematically toward higher Pos(G) values (∼1595 cm^−1^) and increased Pos(2D) positions (∼2695 cm^−1^) (Figure 2C). The concurrent upshift of both peaks together with the narrowing and increased clustering of the data distribution points to substantial p‐type doping via π–π interactions between the graphene and the pyrene group of PBASE [28]. The magnitude of the 2D upshift, which exceeds typical doping‐induced changes, further suggests the presence of a compressive strain component. Analysis of the Pos(2D) against Pos(G) correlation shows a slope lying between the characteristic values for pure strain (∼2.2) and pure doping (∼0.7) [29], confirming that both effects contribute, but with doping as the dominant factor. The observed decrease in the I_2D_/I_G_ ratio from ∼2.5 to ∼1.5 is consistent with increased carrier concentration, showing that PBASE immobilization induces pronounced p‐type doping in graphene, accompanied by a strain contribution. An x‐ray photoelectron spectroscopy (XPS) analysis provided the chemical validation of PBASE adsorption by detecting the presence of nitrogen, which is absent from pristine graphene. A distinct N 1s peak at 400.25 eV was observed in the functionalized sample (Figure 2D), confirming the presence of PBASE. Additionally, the deconvolution of the C 1s spectrum revealed a C─N peak at 285.68 eV, a C─O peak at 286.65 eV, and an O = C─O peak at 288.5 eV, in addition to the primary C─C peak at 284.80 eV. This further supports the conclusion that successful PBASE attachment to the graphene surface has occurred, and the full XPS survey spectrum of CVD‐G/PBASE is presented in Figure S6. Atomic force microscopy (AFM) was employed to further evaluate the thickness of the functionalized layer (Figure 2E), and step‐height measurements revealed that pristine graphene had a thickness of 1.71 ± 0.28 nm, increasing to 2.37 ± 0.20 nm after PBASE functionalization. The difference of 0.66 nm is consistent with a monolayer coverage of PBASE, thus validating successful functionalization.

To quantify the thickness of the functionalization layer and its coverage on graphene, quartz crystal microbalance with dissipation (QCM‐D), *I–V* transfer curves and XPS were employed. QCM‐D detects mass adsorption by monitoring frequency shifts, enabling the precise quantification of surface coverage. It was first used to investigate the functional bio interface by evaluating different PBASE concentrations and solvents (Figure S4). Among widely used conditions [15, 30, 31], 2 mM PBASE in ethanol yielded higher antibody immobilization than 2 mM PBASE in methanol. Based on the antibody mass change obtained by QCM‐D, the corresponding receptor densities were estimated to be 0.014 and 0.009 molecules nm^−2^, respectively. In contrast, varying the PBASE concentration produced only minor differences in antibody immobilization, suggesting that surface coverage approached saturation under these conditions. QCM‐D was then used to calculate the molecular density and thickness of the PBASE functionalization layer on graphene (Figure 2F). The resulting frequency shift of 5 Hz corresponded to a PBASE layer thickness of 1.14 nm, with a molecular density of approximately 1.4 molecules per nm^2^ as calculated using the Sauerbrey equation (Equation S1). Repeating this protocol five times yielded an average density of 1.38 molecules per nm^2^. This aligns with previous reports of ∼1.62 molecules per nm^2^ [15], confirming the reproducibility of our methodology. *I–V* transfer curves provided in Figure S7 were used to calculate the PBASE‐induced charge on the graphene and were estimated by dividing this value by the charge per PBASE molecule. The calculations yielded an estimated molecular density of 1.49 molecules per nm^2^. XPS was used to quantitatively determine the molecular density of PBASE noncovalently adsorbed onto the graphene surface. This quantification was performed using a mass balance model that related the total measured N/C atomic percentage ratio to the known theoretical areal density of monolayer graphene, yielding a molecular density of 1.23 molecules per nm^2^. The details of these molecular density calculations for each method are presented in Sections S7–S9. A systematic comparison using QCM‐D, *I–V* transfer curves, and XPS yielded molecular density estimates of 1.40, 1.49, and 1.23 molecules per nm^2^, respectively, while a theoretical calculation for PBASE on graphene based on a footprint of ∼0.26 nm^2^ per molecule gives a value of ∼3.81 molecules per nm^2^. All of these values fall within the reported experimental range of 0.364–1.62 molecules per nm^2^ [15, 32], and are consistent with those of a realistic monolayer in the presence of defects, steric hindrance, and site heterogeneity.

### 2.2 Antibody Fragmentation and Quantification

**FIGURE 3:**
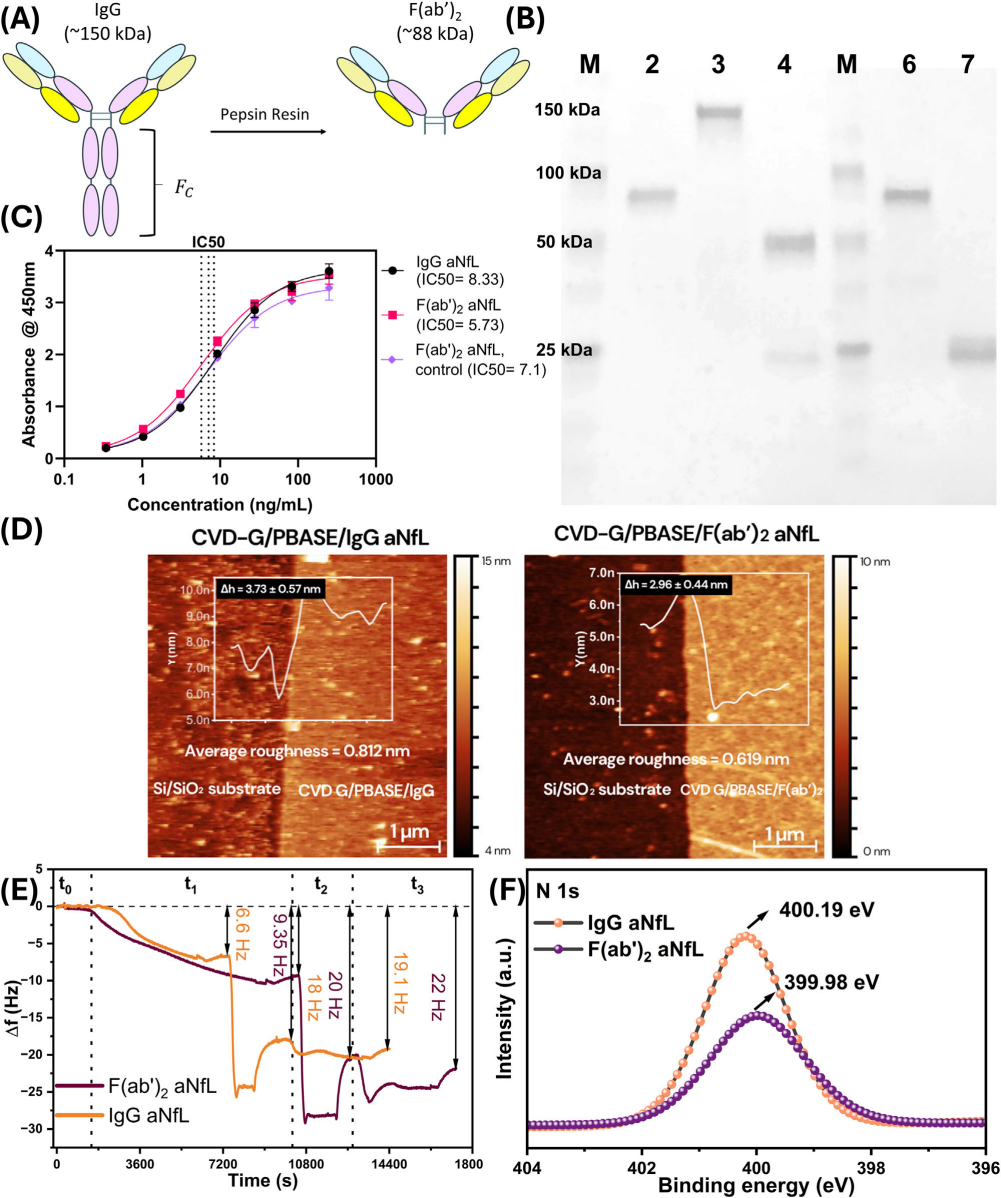
Confirmation of F(ab’)_2_ fragments and their immobilization. (A) Illustration of the structures of the IgG antibody and F(ab’)_2_ fragment. (B) SDS‐PAGE analysis of processed F(ab’)_2_ fragments: Lane 2 contains 1 µg positive control; Lane 3 contains 1 µg non‐reduced IgG antibody; Lane 4 contains 1 µL reduced IgG antibody; Lane 6 contains 1 µg non‐reduced F(ab’)_2_ aNfL; Lane 7 contains 1 µg reduced F(ab’)_2_ aNfL; and Lanes 1 and 5 were used to give an estimation of molecular weight. 2‐Mercaptoethanol, a strong reducing agent, was used to reduce the samples. (C) Indirect ELISA results of IgG aNfL, F(ab’)_2_ and the positive control (*p* = 0.97). (D) Average step height for IgG aNfL and F(ab’)_2_ aNfL immobilization on the graphene surface. (E) QCM‐D was employed to quantify the immobilization of IgG aNfL and F(ab’)_2_ aNfL on the graphene surface. The vertical dashed lines indicate the approximate timeframes for each flow change, while the numerical values represent the final measurements obtained before the next flow step. t_0_: PBS flow 40 µL/min t_1_: 15 nM antibody/PBS flow 10 µL/min t_2_: 2% BSA/PBS flow 40 µL/min. t_3_: 1.85 nM NfL/PBS flow 40 µL/min. (F) XPS spectra of N 1s core for IgG aNfL and F(ab’)_2_ aNfL.

F(ab’)_2_ fragments (F(ab’)_2_ aNfL) were generated from the IgG NfL antibody (IgG aNfL, ab253512), which targets the intermediate filament rod domain of NfL, via an acidic endopeptidase digestion of the Fc domain, as illustrated in Figure 3A. The success of the reaction was confirmed using bicinchoninic acid (BCA), sodium dodecyl sulphate‐polyacrylamide gel electrophoresis (SDS‐PAGE), and an enzyme‐linked immunosorbent assay (ELISA). BCA was employed to determine the final concentration of F(ab’)_2_ fragments (Figure S10), while SDS‐PAGE was used to monitor the extent of digestion. Finally, an indirect ELISA was performed to assess whether or not the fragmentation process affected the binding kinetics compared to unprocessed IgG aNfL antibodies. The SDS‐PAGE results (Figure 3B) confirm successful fragmentation, where Lanes 1 and 5 contained molecular weight markers while Lane 2 served as a positive control showing a successfully digested F(ab’)_2_ fragment (∼88 kDa). Lane 3 containing unprocessed IgG aNfL (∼150 kDa) and Lane 4 representing fully reduced IgG aNfL (∼50 and 25 kDa) served as negative controls to illustrate the start and endpoint of the reaction, respectively. Lane 6 displays the experimental F(ab’)_2_ aNfL (∼88 kDa), and Lane 7 represents the reduced F(ab’)_2_ aNfL (∼25 kDa). The molecular weight comparison validated the successful production of F(ab’)_2_ aNfL. To evaluate the binding kinetics, an indirect ELISA was performed (LoD = 0.105 ng/mL; LoD = Sb + 3σ where Sb is the average value of the blank signal measurement, and σ is the standard deviation of the blank signal measurement) using serial dilutions (starting at 250 ng/mL with a 1:3 dilution series) of unprocessed IgG aNfL, F(ab’)_2_ aNfL, and a successful F(ab’)_2_ aNfL sample as a positive control. As shown in Figure 3C, the nonlinear fit curves for IgG aNfL, F(ab’)_2_ aNfL, and the positive control closely overlapped, suggesting that fragmentation did not affect the binding kinetics. A statistical comparison of the slope values confirmed no significant difference (*p* = 0.9725) between the datasets. For further interpretation, IC50 values were analyzed. This parameter represents the concentration required to inhibit 50% of a biological process, and a lower IC50 indicates a higher binding affinity. The IC50 values for unprocessed IgG aNfL, F(ab’)_2_ aNfL, and the positive control were 8.33, 5.73, and 7.06, respectively, indicating that the fragmentation process did not compromise the antibody binding kinetics. However, further validation on graphene was necessary, prompting additional quantitative analyses using *I–V* transfer curves, QCM‐D and XPS.

Following the immobilization of the probe molecules on the graphene surface, the surface charge introduced by the NfL bioconjugates was estimated from the Dirac‐point shift of the GFET in order to estimate the receptor densities. The charge density induced by binding events was calculated using a planar electric‐double‐layer model, and the value of λ_D_ was calculated based on 0.01× plasma ionic strength, that is, λ_D_ ≈ 7.85 nm. Use of the planar electric‐double‐layer model yielded charge densities of 3.46 × 10^−21^ C nm^−2^ and 5.77 × 10^−21^ for IgG aNfL and F(ab’)_2_ aNfL, respectively. Using the previously reported charge densities per receptor, receptor densities were determined to be 0.0020 molecules nm^−2^ and 0.0084 molecules nm^−2^ for IgG aNfL and F(ab’)_2_ aNfL, respectively (Figure S13). Details of these calculations are provided in Section S15. These findings highlight the structural differences between IgG aNfL and F(ab’)_2_ aNfL, with the smaller F(ab’)_2_ fragment enabling a 4.2‐fold more compact receptor layer on graphene. The higher receptor density observed for F(ab’)_2_ aNfL suggests a greater number of binding sites per unit area, thereby potentially enhancing device performance. Additionally, an AFM step‐height analysis confirmed that the F(ab’)_2_ aNfL layer was positioned closer to the graphene channel than the full IgG construct. The functionalized devices exhibited overall height increases of 3.73 ± 0.57 nm for IgG aNfL and 2.96 ± 0.44 nm for F(ab’)_2_ aNfL (Figure 3D). After subtracting the baseline graphene height, the effective layer thicknesses were 1.36 nm for IgG aNfL and 0.59 nm for F(ab’)_2_ aNfL. The shorter distance for F(ab’)_2_ aNfL places the binding events closer to the graphene channel, reducing screening effects and enabling stronger electrostatic interactions. In contrast, the lower surface packing density and greater distance of IgG aNfL may limit overall device performance. Additionally, the values of surface roughness (R_a_) for CVD‐G/PBASE functionalized with IgG aNfL and F(ab’)_2_ aNfL are shown in Figure S11A. The immobilization of F(ab’)_2_ aNfL exhibited a lower R_a_ (0.423 nm) than that of the IgG aNfL layer (0.694 nm), further indicating the closer proximity of F(ab’)_2_ aNfL in the GFET sensors.

To confirm the conformation of F(ab’)_2_ aNfL on the graphene surface, three‐dimensional AFM topography results are presented in Figure S11B, showing the CVD‐G/PBASE/F(ab’)_2_ aNfL after bovine serum albumin (BSA) blocking and analyte binding. A monotonic rise in surface roughness with each step further supports the successful functionalization of the CVD‐G. QCM‐D was then used to quantify the interaction between antibody and analyte, and the results are shown in Figure 3E. Following antibody immobilization and a PBS wash, frequency shifts of 6.6 Hz and 9.35 Hz were observed for IgG aNfL and F(ab’)_2_ aNfL, respectively. After BSA blocking and subsequent PBS washing, the respective shifts increased to 18 Hz and 20 Hz, respectively. After the subsequent introduction of 1.85 nM NfL for an hour, followed by a final PBS wash, the frequency shifts reached 19.1 Hz for IgG aNfL and 22 Hz for F(ab’)_2_ aNfL. These results indicate that the F(ab’)_2_ fragments enabled approximately 1.5 times more immobilization, probably due to their smaller size, which allowed greater receptor density on the surface. NfL binding further corroborated this effect, yielding frequency shifts of 1.1 Hz for IgG aNfL and 2 Hz for F(ab’)_2_ aNfL, thereby demonstrating an 82% increase in binding events simply by incorporating F(ab’)_2_ fragments. A computational analysis of mass change over time (Figure S12) confirmed these trends. Receptor density was found to be 0.004 nm^−2^ for IgG aNfL and 0.011 nm^−2^ for F(ab’)_2_ aNfL, yielding a 2.75‐fold increase. Similarly, analyte density improved 1.78‐fold, from 0.0014 nm^−2^ to 0.0025 nm^−2^. This nearly threefold enhancement in antibody immobilization naturally expanded binding site availability, resulting in a 78% enhancement in analyte capture and helping to improve the sensitivity of the GFET device. To further support these findings, an XPS analysis of the N 1s core‐level peak was performed, as shown in Figure 3F. The XPS spectra for IgG aNfL and F(ab’)_2_ aNfL show distinct peak intensities at binding energies of 400.19 eV and 399.98 eV, respectively, after C 1s referencing to 284.8 eV. These values of binding energy are well within the range expected for amide‐type nitrogen in proteins (∼400 eV). The slight shift of the F(ab’)_2_ aNfL N 1s peak to a lower binding energy (ΔE ≈ −0.21 eV) could be attributed to the closer proximity of the probe to the graphene surface, resulting in a modest increase in electron density at the nitrogen atom and stronger electrostatic interaction at the protein‐graphene interface. The XPS survey spectra of both CVD‐G/PBASE/IgG aNfL and CVD‐G/PBASE/F(ab’)_2_ aNfL are presented in Figure S11C, while the XPS N 1s spectrum, confirming the successful immobilization of IgG aNfL, is shown in Figure S11D.

### 2.3 Performance of GFET Devices

Electrical measurements were performed on the GFET devices functionalized with unprocessed IgG aNfL so as to establish a baseline performance. NfL was spiked into PBS and human plasma over 0.15–1500 pg/mL. The corresponding transfer curves and signal comparisons are shown in Figure S8. In both matrices, a positive shift in the Dirac point was observed with increased NfL concentration, giving a linear detection range across 0.96–1500 pg/mL. The positive shift in the Dirac point following NfL binding was assumed to be due to the negative charge of NfL (pI = 4.63) [33], which induces p‐doping in the graphene channel. This also suggests that the detection mechanism is primarily governed by electrostatic interactions between the charged analyte and the graphene surface. Notwithstanding this, noticeable signal variability was observed in both matrices, indicating that overall response reproducibility required improvement at this stage.

**FIGURE 4:**
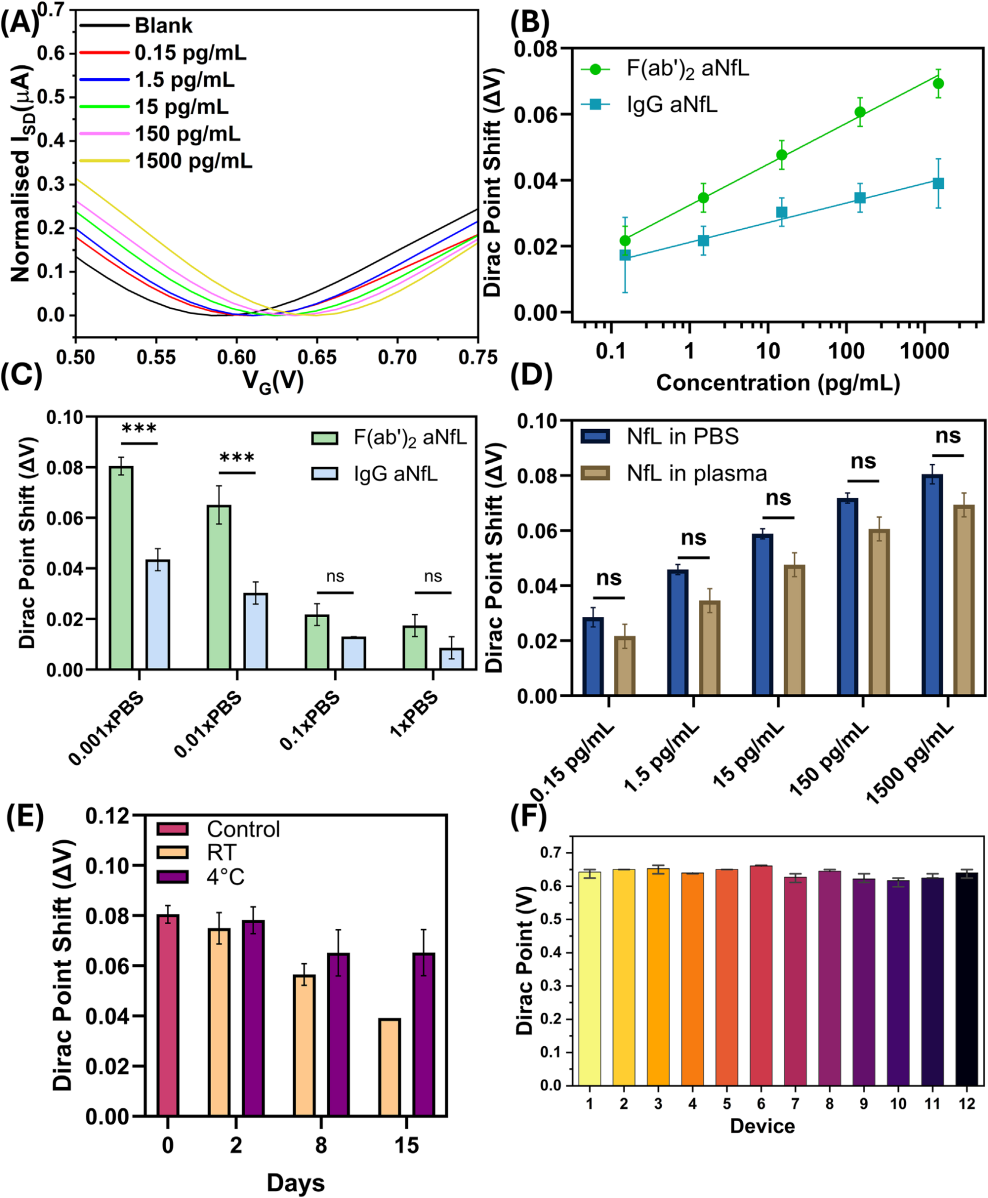
Device performance of F(ab’)_2_ aNfL‐immobilized GFET devices. (A) Transfer curves of GFET in plasma with and without NfL biomarkers. (B) Performance comparison of IgG aNfL‐immobilized and F(ab’)_2_ aNfL‐immobilized GFET devices (*n* = 3, *p* < 0.0001). (C) Dose‐response comparison with varying PBS ionic strengths for both IgG‐ and F(ab’)_2_‐functionalized GFETs (*n* = 3, *p* = 0.001 and *p* = 0.002). (D) Comparison of mean device signal intensity in PBS and plasma (*n* = 3, *p* = 0.2). (E) Potential shelf‐life evaluation of F(ab’)_2_ aNfL‐immobilized GFETs (*n* = 3). (F) Repeatability and reproducibility of 12 GFET devices.

To enhance device performance, the antibody immobilization strategy was modified by replacing whole IgG antibodies with F(ab’)_2_ fragments. Representative *I–V* transfer curves acquired before and after the addition of NfL are shown in Figure 4A. In both PBS and plasma, the Dirac point shifts increased progressively with NfL concentration. A direct comparison of device responses with IgG aNfL‐ and F(ab’)_2_ aNfL‐functionalized GFETs is provided in Figure 4B. While both configurations exhibited a concentration‐dependent response across the same tested range (0.15–1500 pg/mL), the F(ab’)_2_‐functionalised GFETs achieved a considerably better fit. Linear regression analysis yielded R^2^ values of 0.9977 (Y = 0.01267*X + 0.03253) for F(ab’)_2_ aNfL‐immobilized GFETs and 0.9396 (Y = 0.005924*X + 0.02197) for IgG aNfL‐immobilized GFETs. The sensitivity (slope, S) of the F(ab’)_2_ aNfL‐immobilized GFETs was enhanced by 114% relative to IgG‐immobilized devices, indicating a stronger output per unit change in analyte concentration. The mean coefficient of variation (CV%) for F(ab’)_2_ aNfL‐immobilized devices (17.7%) was 2.3‐fold lower than that of IgG‐immobilized devices (41.5%), indicating substantially reduced response variability. LoD values were determined according to Equation (2), using the standard deviation of the response (Sy) and the slope of the calibration curve (S):

(2) $$LoD=3.3\left(\frac{s_{y}}{s}\right)$$

The LoD was found to be 0.18 pg/mL for F(ab’)_2_ aNfL‐immobilized GFETs and 0.96 pg/mL for IgG aNfL‐immobilized GFETs, corresponding to a 5.3‐fold improvement when using F(ab’)_2_ fragments. These improvements are consistent with the expected mechanism, in which the positioning of negatively charged NfL molecules closer to the graphene surface places the binding event within the Debye length, reducing ionic screening effect and enhancing the transduced binding signal. In order to directly investigate this effect, device responses were measured for F(ab’)_2_ aNfL‐ and IgG aNfL‐immobilized GFETs at a fixed NfL concentration across PBS concentrations ranging from 0.001x to 1x (Figure 4C). For both receptors, the response decreased monotonically with increasing ionic strength, which is consistent with stronger Debye screening at higher ionic strength. At 0.001x and 0.01x PBS, F(ab’)_2_ aNfL‐immobilized GFETs exhibited significantly greater responses compared to their IgG counterparts (*p* = 0.001 and *p* = 0.002, respectively), whereas at 0.1× and 1× PBS the responses were small and not statistically different between the two receptor types. These observations reflect the interaction between the Debye length and receptor dimensions. At 0.001x PBS (λ_D_ ≈ 23 nm), both receptor‐analyte complexes lie within the Debye length; however, electrostatic coupling to the graphene channel still decays with distance, resulting in more efficient charge transduction for the shorter F(ab’)_2_ fragments. At 0.01x PBS (λ_D_ ≈ 7.3 nm), F(ab’)_2_ receptors remain mostly within the Debye length while IgG‐bound analytes are predominantly screened, leading to a pronounced difference in response. At 0.1x and 1x PBS (λ_D_ ≈ 2.3 and 0.7 nm, respectively), both receptor‐analyte complexes are effectively screened, yielding minimal and indistinguishable responses. These results indicate that a reduction in receptor size enhances effective electrostatic coupling primarily under intermediate screening conditions, while operation at physiological ionic strength will require additional strategies to mitigate Debye screening for clinical deployment. Real‐time measurements were conducted to assess the dynamic response of the device (Figure S9A). Switching from PBS to healthy plasma initially altered the I_SD_ current; however, it returned to approximately the same I_SD_ current within the same time interval. Upon the addition of 0.15 pg/mL NfL, a decrease in the value of I_SD_ was observed within the same time interval. This concentration in Figure S9A represents the lowest concentration tested experimentally, whereas the LoD (0.18 pg/mL) was determined as a statistically defined threshold, as discussed below. As such, signals can still be measured just below this value, but with increased uncertainty. The small difference between 0.15 pg/mL and the calculated LoD lies well within the expected variability associated with sample preparation, device response, and statistical estimation near the detection limit. As shown in Figure 4D, no statistically significant matrix effect on device performance was observed between PBS (CV = 8.33%) and plasma (CV = 17.7%) (*p* = 0.2, two‐way ANOVA with Šídák's multiple‐comparisons test), although the higher variability in plasma may reflect matrix‐related effects. Other factors, such as device‐to‐device variation and the surface functionalization process, may also contribute to the observed variability. In our experiments, device‐to‐device variability in carrier mobility within a single wafer was approximately 20%, reflecting differences introduced during CVD growth, and transfer and fabrication processes. In addition, the NHS‐amine coupling chemistry used for F(ab’)_2_ immobilization does not provide strict control over fragment orientation, leading to differences in antigen‐binding site accessibility. Given the high sensitivity of GFETs to local surface charge modulation, such small variations can translate into measurable signal differences. Future improvements may be achieved through alternative and more controlled functionalization strategies, such as π‐π stacking‐based linker systems, such as the tetrakis (4‐carboxyphenyl) porphyrin or electric‐field‐mediated alignment of antibodies [34, 35, 36]. These approaches could improve surface uniformity and antibody orientation, thereby reducing variability while preserving the enhanced sensitivity observed for the F(ab’)_2_ platform. In addition to the evaluation of the effect of matrix, cross‐reactivity with structurally related neurodegeneration biomarkers (GFAP, Tau316, and pTau217) was also tested (Figure S9B). The F(ab')_2_ aNfL‐immobilized GFETs showed negligible responses, confirming high specificity for NfL. The potential shelf‐life and stability of the GFET platform were investigated next. By considering realistic deployment conditions, the F(ab’)_2_‐immobilised GFETs were stored dry without antigen at room temperature or at 4°C for up to 2 weeks before the introduction of NfL. As shown in Figure 4E, no significant decline in sensor response was observed up to 2 days in both conditions; however, at 1 week, sensor response declined by 30% when it was stored at room temperature, while storage at 4°C retained 80% of the response. At week 2, the response decreased by 50% for room temperature storage, whereas storage at 4°C was still capable of retaining 80% of the response, suggesting that the immobilized antibodies remain mostly stable and functionally active for at least 2 weeks under dry storage at 4°C. These findings support the feasibility of the short‐term storage and distribution of pre‐functionalized GFETs for POC applications; however, extended shelf‐life investigations should be carried out, and several strategies to enhance the stability of immobilized antibodies on solid surfaces, such as the addition of vinylsulfonated‐polyethyleneimine nanoscaffold, PEG layer or protective zeolitic imidazolate framework‐8 coating [37, 38, 39, 40], could be employed to increase the potential of GFETs for practical POC deployment. Repeatability was examined for twelve GFETs exposed to 15 pg/mL NfL in PBS with measurements taken five times (Figure 4F). The Dirac point remained stable with a CV of 2.12%, indicating high repeatability under identical conditions. Overall, F(ab’)_2_ functionalization improved sensitivity and precision while maintaining robust performance in plasma, supporting its suitability for clinical applications.

### 2.4 Clinical Sample Measurements With GFET and Simoa

**FIGURE 5:**
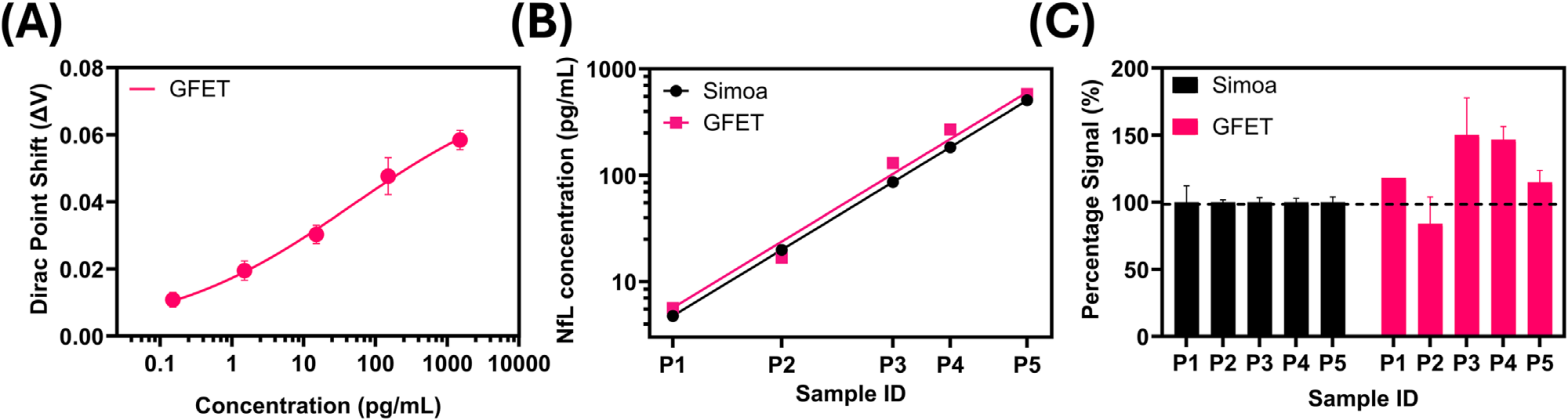
Detection of NfL in clinical plasma samples using the GFET platform. (A) Standard curve generated in spiked human plasma for GFET‐based NfL detection (*n* = 6). (B) Correlation analysis of NfL concentrations measured in five patient plasma samples using Simoa and GFET (*p* = 0.0007). (C) Recovery rate of NfL concentrations measured by GFET in comparison to Simoa as a reference.

The clinical samples collected were labelled with sample IDs from P1 to P5 and quantified using a standard curve generated from spiked human plasma (0.01x). The dilution step is simple and rapid, requiring only the brief addition of plasma to preprepared buffer, without extensive sample processing or buffer exchange. This approach balances the physical requirements for GFET sensitivity with the operational simplicity and speed required for POC applications. The curve was established by dose‐response fitting and exhibited a clear linear relationship (R^2^ = 0.99) as shown in Figure 5A. Each sample was tested in six replicates, yielding interpolated concentrations ranging from 6 to 581 pg/mL with an average CV of 14.46%, demonstrating the GFET platform's acceptable level of repeatability. To benchmark the GFET results against a clinically validated method, NfL levels were also measured using the Simoa assay. The Simoa results ranged from 5 to 506 pg/mL with CV values below 5% for all samples except P1, which exhibited a CV of 12%. The reported LoD for Simoa is 0.071 pg/mL [41], confirming its high analytical sensitivity and precision. Both techniques consistently identified P1 as having the lowest and P5 as the highest NfL concentrations, indicating strong qualitative agreement between the two platforms. A quantitative comparison of the interpolated data revealed a strong linear correlation between the GFET and Simoa measurements (Figure 5B), with a Pearson correlation coefficient of r = 0.99 (*p* = 0.0007). Additionally, the recovery analysis (Figure 5C) further supported this agreement, showing an average recovery rate of 123% relative to Simoa. This likely reflects systematic differences between the platforms rather than any intrinsic signal amplification by the GFET. In GFET‐based sensing, antigen binding modulates the local surface potential, producing electrical signal changes governed by the effective charge distribution of the bound proteins, whereas Simoa relies on enzyme‐mediated fluorescence that is largely independent of protein charge. Given these fundamentally different detection mechanisms, calibration differences between GFET and Simoa measurements, small device‐to‐device variability, and the limited number of clinical samples (*n* = 5) can contribute to systematic deviations. Furthermore, NfL may exhibit structural heterogeneity and microheterogeneity in net charge arising from sequence composition, conformational variability, and post‐translational modifications, which can vary between individuals and across disease states [42, 43]. These variations in effective surface charge may influence the magnitude of the electrical response measured by GFETs, whereas Simoa signals are largely unaffected by such differences. Despite these structural distinctions, both platforms exhibited a consistent concentration‐dependent trend, validating the quantitative reliability of the GFET biosensor for plasma NfL detection. Detailed interpolated concentrations from each platform are provided in Table S2.

The high correlation between the two methods suggests that the GFET platform could be a cost‐effective, rapid, and accurate platform for the detection of dementia biomarkers, offering a valuable tool for the early diagnosis and monitoring of neurodegenerative diseases. Additionally, the LoD improvements associated with the GFET platform are evident when compared to other detection methods for NfL levels (Table 1). Overall, the strong agreement observed with Simoa underscores the potential of the proposed platform. Nevertheless, it is important to note that this comparison was conducted using five clinical plasma samples. Therefore, validation in a larger cohort would be required to robustly establish the clinical performance of the proposed platform.

**TABLE 1:** LoD comparison of platforms for NfL detection.

Platform	Recognition element	Matrix^a^	LoD	Refs.
GFET	F(ab’)_2_ aNfL	Plasma	0.18 pg/mL	This work
GFET	IgG aNfL	Plasma	0.96 pg/mL	This work
Fluorescent Assay (CRISPR‐Cas12a)	IgG aNfL	Buffer (NaCl, PBS, BSA, Tween 20)	0.012 pg/mL	[44]
Photocathodic immunosensor	IgG aNfL	PBS	0.04 pg/mL	[45]
GFET	IgG aNfL	PBS	0.055 pg/mL	[46]
Digital immunoassay (Simoa, HD‐X)	IgG aNfL	Serum	0.071 pg/mL	[41]
Electrochemical sensor	IgG aNfL	Tris‐HCl (with H_2_O_2_)	0.1 pg/mL	[47]
Automated immunoassay (Ella, Simple Plex)	IgG aNfL	Serum	1.09 pg/mL	[41]
Automated immunoassay (Siemens ADVIA)	IgG aNfL	Serum	1.49 pg/mL	[48]
Fluorescent immunosensor	IgG aNfL	Phosphate buffer	1.95 pg/mL	[49]
EGOFET	IgG aNfL	PBS	∼2 pg/mL	[50]
Electrochemical immunosensor	IgG aNfL	Phosphate buffer	3.0 pg/mL	[51]
Electrochemical immunosensor	IgG aNfL	Serum	5.22 pg/mL	[52]
Photoelectrochemical immunosensor	IgG aNfL	PBS	7 pg/mL	[53]
SERS lateral flow assay	IgG aNfL	Plasma	17.38 pg/mL	[54]

^a^ Matrix indicates the calibration medium used to calculate the reported LoD.

## 3 Conclusions

In this work, we have demonstrated an ultrasensitive GFET platform for the detection of NfL as a blood biomarker. By addressing the primary limitations of GFET technology, we have developed a highly sensitive detection platform that approaches the analytical sensitivity of the gold‐standard Simoa platform but without the associated high cost or time requirements. The device performance improvements were primarily achieved through the use of F(ab’)_2_ fragments, which mitigate Debye screening and enhance sensitivity, supported by quantitative characterization of PBASE surface coverage to support controlled antibody immobilization. The F(ab’)_2_‐modified GFET devices successfully detected NfL concentrations in the sub‐picogram per milliliter range, achieving a LoD of 0.18 pg/mL. Given that reported NfL levels in blood typically range from below 5 pg/mL in healthy individuals to approximately 1000 pg/mL in severe pathological conditions [55, 56, 57], our GFET platform demonstrates strong potential as a versatile diagnostic and prognostic tool.

## 4 Experimental Section

### 4.1 Materials and Instruments

Single‐side poly (methyl methacrylate)‐coated CVD‐G and GFET‐S20 were acquired from Graphenea, Spain. 300 nm p‐type 𝑆𝑖𝑂_2_/𝑆𝑖 substrates for GFET fabrication were sourced from Graphene Supermarket, USA. The photoresists LOR 3A and S1805, MF319 developer, Remover SU‐8 2002 negative photoresist and SU‐8 developer were acquired from the London Centre for Nanotechnology facilities. QSX 303 sensors for QCM were obtained from Biolin Scientific, Sweden. The NfL antibody pair (ab253512), human NfL protein 81 kDa (ab224840), structurally related neurodegeneration biomarkers GFAP (ab151370), Tau316 (ab151872) and pTau217 (ab318936) were purchased from Abcam, UK. Laemmli buffer, stain‐free precast gels and unstained protein standards were purchased from Bio‐Rad, UK. The BCA protein assay kit, protein A‐agarose, and immobilized pepsin were purchased from Thermo Fisher, UK. Ammonium persulphate, acetic acid, isopropanol, PBASE, EtOH, methanol, dimethylformamide, PBS tablets (0.01 M phosphate and 0.137 M sodium chloride at pH 7.4) and healthy plasma were sourced from Sigma‐Aldrich, UK. All chemicals were of analytical grade unless specified otherwise.

### 4.2 Fabrication and Characterization of the GFET Device

The GFET comprised a compact sensor array of twelve individual devices separated into two regions by a central Cr/Au gate electrode. The carrier mobilities of individual GFETs, as determined from the transfer curves and applying back gates, are on average 1700 cm^2^ V/s with a standard deviation of σ = 280 cm^2^ V/s determined for 10 batches of devices [58]. A detailed step‐by‐step fabrication protocol is provided in Section S3, along with an illustration of the photolithography process (Figure S3).

SEM was performed to analyze the surface morphology and structure of bare graphene and PBASE‐functionalized graphene samples. The samples were mounted on aluminum stubs using carbon tape, and the characterization was performed using a Gemini (Hitachi S‐3400n, Tokyo, Japan) at a 1 kV electron high tension. An Asylum MFP‐3D Classic AFM and a Bruker Innova system were used to observe the morphology of the graphene surface before and after PBASE functionalization in AC tapping mode. SCOUT 70 probes with an average tip radius of 15 nm were used for scanning at a typical mapping size of 512 × 512 pixels. For the quantitative analysis of surface roughness, thickness and height, three to five images were acquired for each sample and subsequently processed using Gwyddion image analysis software. XPS characterization was conducted using a K‐Alpha+ system equipped with an Al Kα radiation source (hv = 1486.6 eV). The experiments were performed under a base vacuum pressure of 5 × 10^−8^ mbar so as to ensure optimal conditions for spectroscopic analysis. Raman spectra and spatial Raman measurements were acquired using a Renishaw inVia confocal scanning microscope with a 515 nm green laser as the excitation source and an 1800 L mm^−1^ grating. To provide statistical analysis of graphene quality and doping levels, 10 × 10 µm areas were scanned at a step size of 0.1 µm.

Electrical measurements (I_sd_‐V_g_) were performed using an EverBeing C4 probe station interfaced with a Keysight B1500 semiconductor analyzer in 0.001x PBS solution (d1000 PBS, ∼0.15 mM ionic strength) or 0.01x plasma (d100 plasma, ∼1.5 mM ionic strength). During these measurements, the source‐drain voltage was held at 10 mV while the electrolyte gate voltage was swept from 0 to 1 V at a rate of 13 mV/s. NfL‐spiked samples were prepared through a 10‐fold serial dilution of the stock solution in either d1000 PBS or d100 plasma. The samples were incubated on GFETs for 30 min, followed by three washes with d1000 PBS prior to measurements. For measurements in clinical plasma samples, 10–15 µL of the sample was directly applied to the GFETs. The same incubation and washing protocol used for the NfL‐spiked samples was followed before measurements. Additionally, real‐time response measurements were conducted in assessing binding kinetics, as they provide crucial information about the optimal binding time between analyte and probe molecules. The gate voltage in this case was held at 800 mV so as to ensure a high value of transconductance [59]. The complete workflow from sample preparation to final readout required approximately 45 min, comprising plasma sample preparation (1–2 min), incubation (30 min), washing steps (3 min), and electrical measurement with data analysis (10 min).

### 4.3 Antibody Fragmentation and Immobilization

The fragmentation process involves the use of immobilized pepsin (Thermo Fisher Scientific Pierce Immobilized Pepsin, Cat. No. 20343), which is an acidic endopeptidase covalently coupled to crosslinked agarose beads, to cleave the Fc region of IgG aNfL below the hinge region to obtain F(ab’)_2_ fragments. IgG aNfL solution was loaded onto 0.1 M sodium acetate buffer to adjust the pH to 4.4 and then mixed with agarose‐conjugated peptidase for 90 min at 37°C with shaking. The reaction was stopped by cooling the reaction tube to room temperature, followed by centrifuging at 5000 × g for 1 min to separate the antibody fragments and unprocessed antibodies from the agarose‐conjugated peptidase. Then, the F(ab’)_2_ fragments were collected and washed with 1x PBS buffer to adjust the pH to 7.2 in order to facilitate the separation of F(ab’)_2_ fragments. Protein‐A purification with 1x PBS buffer was subsequently performed to recover the F(ab’)_2_ fragments, and the final volume was adjusted to 100–150 µL using 30 kDa threshold concentration columns. The sample concentration was determined via BCA assay, aliquoted to a mass of 5 µg, and stored at −80°C. An indirect ELISA was conducted to confirm the bioactivity of the resulting fragments. SDS‐PAGE was used to verify purity and size by comparing the molecular weight of the obtained fragment (∼88 kDa). The quality controls, including BCA, indirect ELISA, and SDS‐PAGE, are comprehensively detailed in Section S12.

Following the functionalization of the graphene surface with 2 mM PBASE in EtOH for 2 h and subsequent washes with EtOH, 10 µg/mL NfL antibody or F(ab’)_2_ was prepared in 1xPBS and incubated overnight at 4°C. The sample was then washed three times with 1x PBS. A 2% BSA solution was used as a blocking agent and incubated for 1 h at room temperature, followed by three additional washes with 1xPBS. For GFET shelf‐life experiments, functionalized devices were kept dry either at room temperature or 4°C until their test time; for the repeatability experiments, devices were stored in vacuum‐sealed packages to prevent exposure to air until use.

### 4.4 QCM‐D Measurements for Immobilization Confirmation and Quantification

QCM‐D monitoring was performed using a QSense Analyzer from Biolin Scientific. Monolayer CVD‐G was wet‐transferred onto the surface of the QSX 303 sensor following the protocol detailed and illustrated in Figure S2 so as to ensure the full coverage of the active sensing area of quartz microcrystal (∼7.85 × 10^−5^ m^2^) with graphene. For the characterization of PBASE immobilization, at time 𝑡_0_, EtOH was allowed to flow over the QSX 303 sensor surface, then changed to a 2 mM PBASE EtOH solution at time 𝑡_1_. A decrease in the resonance frequency was expected due to the adsorption of the PBASE onto graphene. At time 𝑡_2_, EtOH was allowed to flow over the sensor in order to remove any unabsorbed PBASE. For the characterization of antibody‐antigen binding, graphene‐transferred sensors were functionalized with PBASE prior to each measurement. Mass calculations based on the molecular weights of IgG aNfL and F(ab’)_2_ aNfL were performed to ensure that equal antibody mass was allowed to flow over the sensor (detailed calculations are given in Section S14). A final antibody concentration of 15 nM was used for both types. Durations of reagent flow, including of PBS, antibody, 2% BSA, and NfL, were kept constant except when additional time was required for frequency stabilization.

### 4.5 Clinical Samples and Simoa Cross‐Validation

Participants diagnosed with moderate to severe TBI based on Mayo classification criteria were included in the BIO‐AX‐TBI study (Trial registration number: NCT03534154), which was ethically approved by the Health Research Authority (17/LO/2066). All human plasma samples were collected after acquiring written informed consent from all participants. Eligible participants were 18 to 80 years old at the time of injury and were screened to exclude those with prior neurological illnesses, a history of hospitalization for previous TBI, substance abuse, or pregnancy. Blood was collected from each patient and placed in ethylenediaminetetraacetic acid‐coated tubes for plasma extraction. After 30 min at room temperature, the samples were centrifuged at 2500 × g at 4°C. The plasma was divided into 1.4 mL aliquots and stored at −80°C.

The Simoa HD‐X Analyzer and the NfL Discovery Kit from Quanterix were used to measure NfL levels in plasma according to the supplier's suggested protocol. Plasma samples were initially loaded undiluted and then diluted fourfold within the apparatus. Each sample was treated for 35 min with magnetic beads containing capture antibodies and biotinylated detection antibodies. This was followed by a 5‐min incubation with streptavidin‐β‐galactosidase. The beads were cleaned and reintroduced into a solution containing resorufin β‐D‐galactopyranoside, resulting in optical signal development.

### 4.6 Statistical Analysis

For the quantification of NfL in spiked buffer samples prepared in PBS or healthy plasma using GFET, linear regression was applied to five calibration points. In clinical plasma samples, NfL concentrations were determined through four‐parameter sigmoidal curve fitting, with five standard points used for GFET and seven for Simoa measurements. ELISA data for antibody fragment binding kinetics were also evaluated using a four‐parameter sigmoidal model with seven calibration standards. All values were reported as mean ± standard error of the mean (SEM), with error bars indicating the SEM between replicates. The figure legends indicated the sample sizes (n) for each dataset. Statistical significance was determined using two‐way analysis of variance, and *p*‐values less than 0.05 were considered statistically significant. Non‐significant results were denoted as “ns.” All analyses were performed using Origin2024b or GraphPad Prism 10 software.

## Author Contributions

S.B.K. contributed to conceptualisation and methodology, performed the experiments, carried out data analysis, curation, and validation, contributed to visualisation, and wrote the original draft of the manuscript. R.A. contributed to the experiments, data analysis and editing the manuscript. L.S. contributed to investigation, methodology, and data validation. S.G.E. and A.N.M.M. contributed to investigation and data analysis. A.S., N.N., Y.W., N.G., M.E., A.L., E.T., and H.Z. provided resources and contributed to data interpretation. A.H. contributed to investigation, data curation and provided resources. S.R. contributed to methodology development and data validation. D.J.S. acquired funding, provided resources, and contributed to data interpretation. B.L. supervised the project, contributed to conceptualisation and methodology, and provided project administration and funding acquisition. All authors discussed the results and contributed to the final version of the manuscript.

## Conflicts of Interest

The authors declare no conflicts of interest.

## Supporting information




**Supporting File**: smll73928‐sup‐0001‐SuppMat.docx.

## Data Availability

The data that support the findings of this study are available from the corresponding author upon reasonable request.
