# Supplementary material for: Ultrasensitive Detection of Neurofilament Light in Plasma Using F(Ab’)2‐Modified Graphene Field‐Effect Biosensor
Source: Small. 2026 May 26;22(39):e73928. doi: 10.1002/smll.73928 (PMC13360647; doi:10.1002/smll.73928)
Supplement: Supplementary file 1 — Supporting File: smll73928‐sup‐0001‐SuppMat.docx. [file SMLL-22-e73928-s001.docx]

**Supporting Information**

**Ultrasensitive Detection of Neurofilament Light in Plasma Using F(ab’)_2_-modified Graphene Field-effect Biosensor**

*Selvinaz Burcu Kizilates,^1,Ϯ^ Rica Asrosa,^1,2,Ϯ^ Lenart Senicar,^1,3^ Anuja Sharma,^1^ Seda Gungordu Er,^4^ Nisha Naeem,^5^ Ahmad Nizamuddin bin Muhammad Mustafa,^6,7^ Yang Wu,^8^ Neil Graham,^9,10^ Amanda Heslegrave,^3,11^ Mohan Edirisinghe,^4^ Antonio Lombardo,^5,12^ Elias Torres,^13^ Henrik Zetterberg,^3,11,14,15,16,17,18,19^ Sami Ramadan,^1,6^, David J. Sharp,^9,10^ Bing Li,^1,9,10,*^*

^1^ Institute for Materials Discovery, University College London, London, WC1E 7JE, UK.
^2^ Department of Physics, Universitas Sumatera Utara, Medan, 20155, Indonesia.

^3^ UK Dementia Research Institute, University College London, London, WC1E 6BT, UK.

^4^ Department of Mechanical Engineering, University College London, London, WC1E 7JE, UK.

^5^ Department of Electronic and Electrical Engineering, University College London, London, WC1E 7JE, UK.

^6^ Department of Materials, Imperial College London, London, SW7 2AZ, UK.

^7^ FTKEK, Universiti Teknikal Malaysia Melaka, 76100, Malaysia.

^8^ Electrical Engineering Division, University of Cambridge, Cambridge, CB3 0FA, UK.

^9^ Department of Brain Sciences, Imperial College London, London, W12 0BZ, UK.

10 Care Research and Technology Centre, UK Dementia Research Institute, London, W12 0BZ, UK.

^11^ Institute of Neurology, University College London, London, WC1E 6BT, UK.

^12^ London Centre for Nanotechnology, 19 Gordon St, London WC1H 0AH, UK.

^13^ Graphenea Semiconductor, Paseo Mikeletegi 83, San Sebastián, 20009, Spain.

^14^ Department of Psychiatry and Neurochemistry, University of Gothenburg, Mölndal, 43141, Sweden.

^15^ Clinical Neurochemistry Laboratory, Sahlgrenska University Hospital, Mölndal, 43141, Sweden.

^16^ Hong Kong Centre for Neurodegenerative Diseases, Hong Kong, 999077, China.

^17^ Centre for Brain Research, Indian Institute of Science, C.V. Raman Avenue, Bangalore, 560 012, India.

^18^ Department of Pathology and Laboratory Medicine, School of Medicine and Public Health, University of Wisconsin, Madison, Wisconsin, WI 53792, USA.

^19^ Wisconsin Alzheimer’s Disease Research Center, School of Medicine and Public Health, University of Wisconsin, Madison, Wisconsin, WI 53792, USA.

^Ϯ^ These authors contributed equally.

*Corresponding author*.*

E-mail: [bing.li@ucl.ac.uk](mailto:bing.li@ucl.ac.uk)

# **1. Optical Image of the GFET Device**

Figure S1 illustrates the device architecture of the GFET platform, which comprises source (S) and drain electrodes (D), the graphene channels, and an on-chip gate electrode (G) to enable liquid gating.


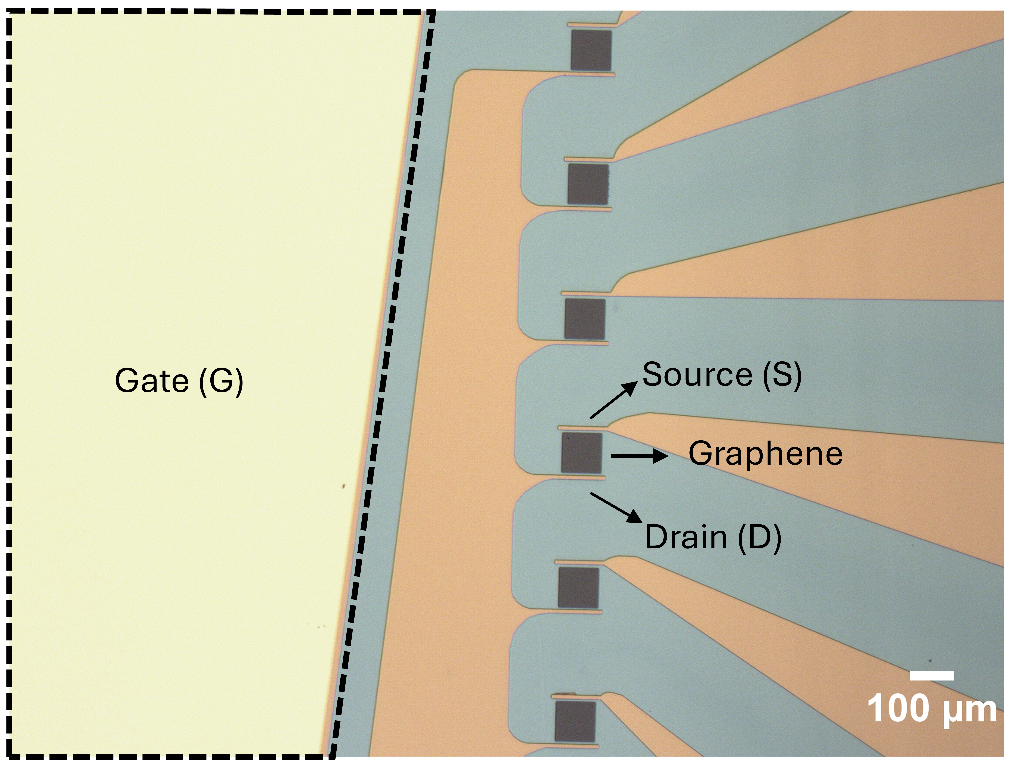


**Figure S1.** Optical image of the GFET device with labelled features. Source and drain electrodes and the graphene channel are indicated with arrows, while the gate electrode is indicated with a dashed outline. Scale bar: 100 µm.

# **2. Wet Transfer of CVD Graphene**

Poly (methyl methacrylate) (PMMA)-coated chemical vapour deposited graphene (CVD-G) on copper (Cu) foil was placed in a 0.1 M aqueous ammonium persulphate (APS) solution to etch the Cu foil. Once the Cu was fully dissolved, the floating PMMA/graphene stack was transferred through multiple washes in deionised (DI) water to eliminate any residual ions. The cleaned graphene stack was then transferred onto a silicon substrate with a silicon dioxide (SiO_2_/Si) substrate or a QSX 303 sensor by scooping it from the water surface. The PMMA/graphene/substrate stack was left to dry at room temperature to remove any trapped DI water droplets between the graphene and the substrate. Following natural drying, the sample was annealed at 180°C for 30 minutes. After cooling, the PMMA coating was removed by immersing the sample in acetic acid overnight, followed by a final rinse in isopropyl alcohol (IPA). The complete transfer protocol is illustrated in Figure S2.


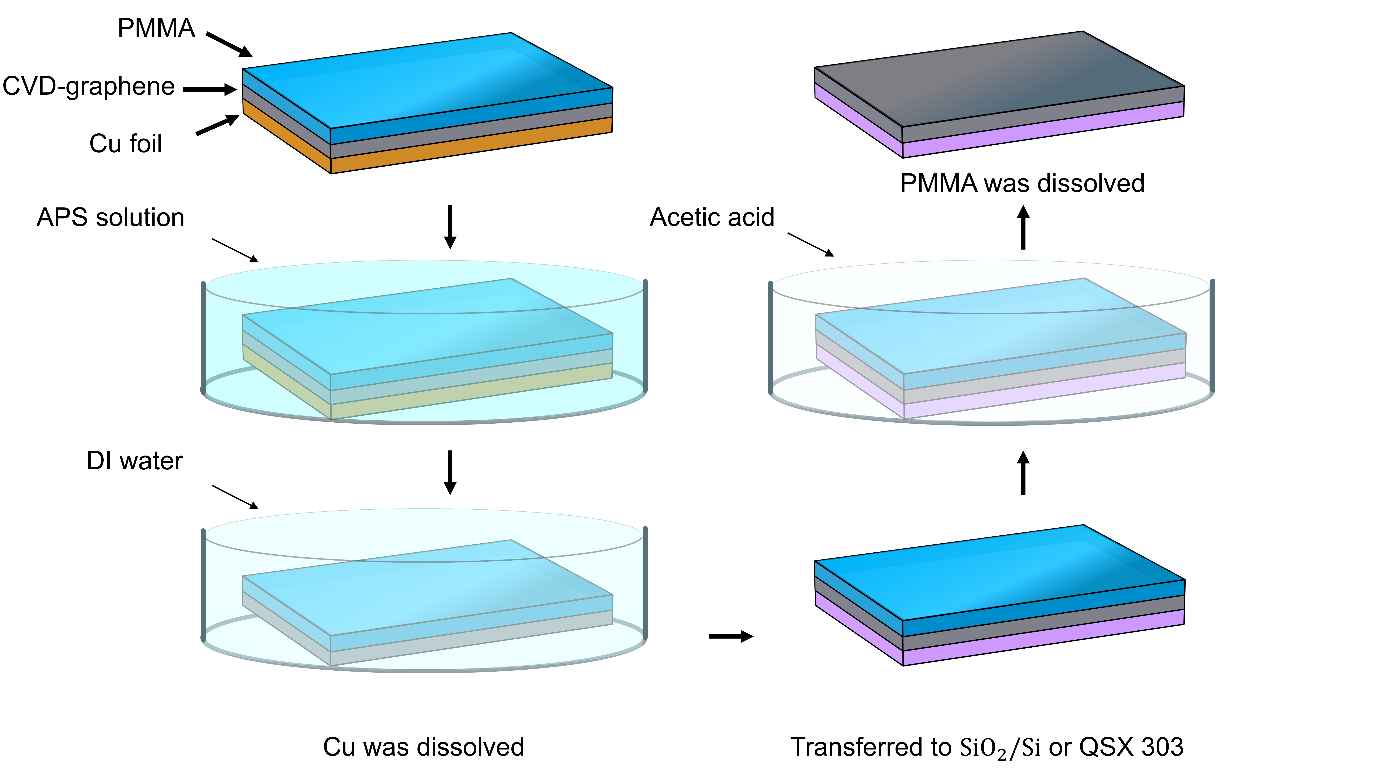


**Figure S2.** Schematic of CVD-G wet-transfer process.

**3. Fabrication of Graphene Field-Effect Transistors (GFETs)**

SiO_2_/Si wafers were initially sonicated in acetone for 20 minutes, then rinsed with IPA and dried with a nitrogen gun. Oxygen plasma treatment (Diener Asher, 5 minutes, full power) was applied to remove residual contaminants. CVD-G was then transferred onto SiO_2_/Si substrates using a PMMA-assisted wet transfer method as described previously. An illustration of the step-by-step photolithography process is provided in Figure S3S3. First, an S1805 photoresist layer was spin-coated at 3500 rpm for 10 seconds and soft-baked at 115 °C for 1 minute (Figure S3A). The sample was then exposed to UV light using a Quintel mask aligner for 7 seconds to pattern the graphene channels (Figure S3B) and developed in MF319 for 1 minute (Figure S3C), followed by rinsing with deionised water and nitrogen drying. Graphene was patterned by oxygen plasma treatment (Figure S3D), and the photoresist was removed by lift-off (Figure S3E). For electrode patterning, an LOR layer was spin-coated at 4000 rpm for 30 seconds and post-baked at 175 °C for 5 minutes, followed by the application of an S1805 photoresist layer (Figure S3F). The sample was again exposed to UV light for 7 seconds (Figure S3G) and developed in MF319 (Figure S3H). A Cr/Au (5 nm/50 nm) layer was deposited via electron beam evaporation (Figure S3I), followed by a two-step lift-off process in 1165 remover at 70 °C for 5 minutes each. Finally, an SU-8 2002 passivation layer was spin-coated, soft-baked at 95 °C for 3 minutes, UV-exposed for 10 seconds, post-baked at 95 °C for 2 minutes, and developed in SU-8 developer for 1 minute, followed by an IPA rinse (Figure S3J).


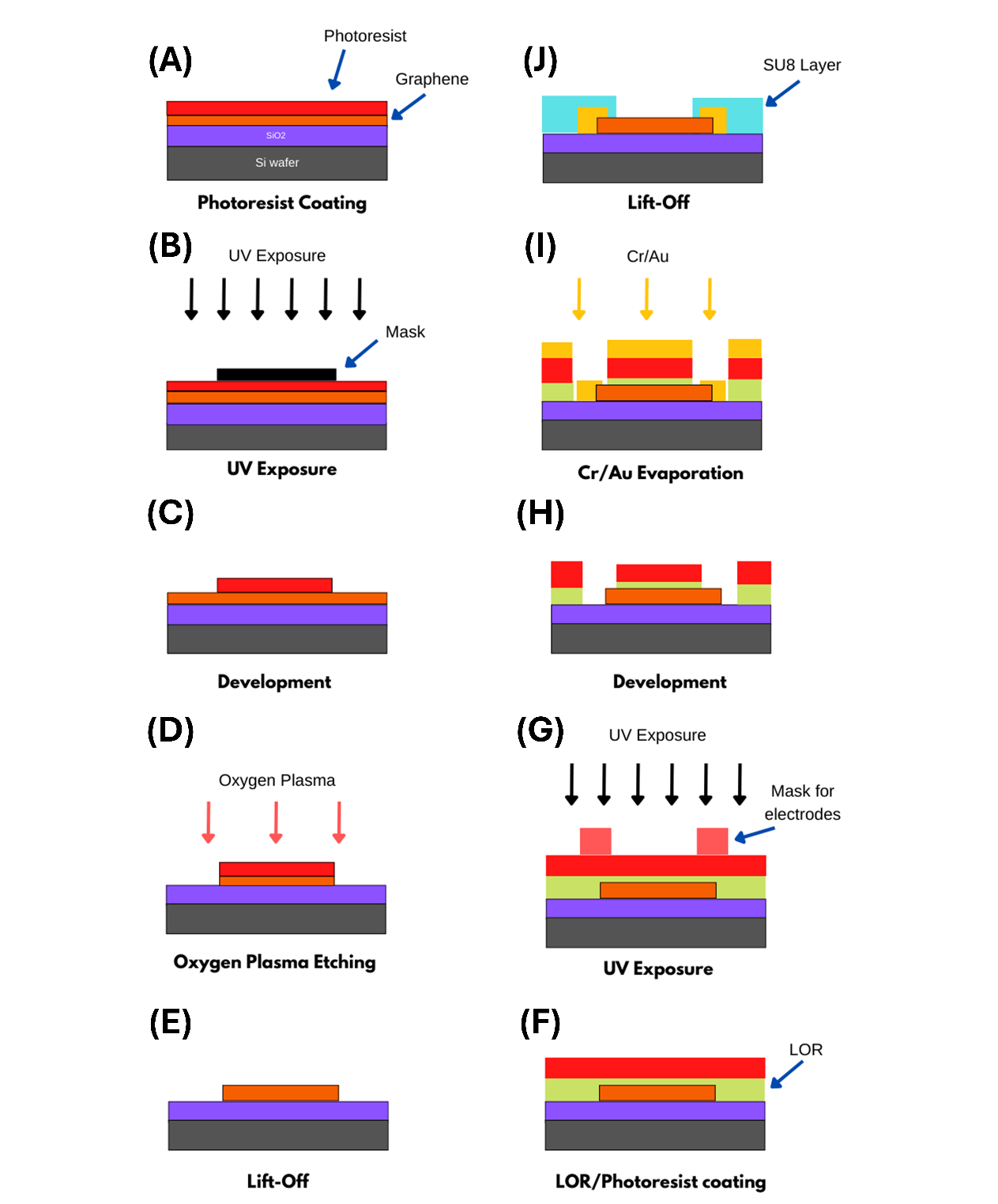


**Figure S3.** Illustration of the step-by-step photolithography process for device fabrication.

# **4. 1-Pyrenebutyric Acid N-Hydroxysuccinimide Ester (PBASE) Characterisation**

To ensure maximum PBASE coverage on the graphene layer, different solvents and concentrations of PBASE solutions were evaluated using quartz crystal microbalance with dissipation (QCM-D). Ethanol (EtOH), methanol, and dimethylformamide (DMF) were initially considered as solvents; however, DMF was excluded from further experiments due to its aggressive nature which caused the degradation of the QCM-D tubing and other plastic components. Additionally, previous studies have shown that DMF reduces the carrier mobility of graphene and induces an n-doping effect.^[1]^ While DMF is an effective solvent for PBASE, its undesirable effects on graphene made it unsuitable for this study. The performance of 2 mM PBASE in EtOH and 2 mM PBASE in methanol was then investigated (Figure S4A). While QCM-D measurements initially indicated a 2.5-fold greater frequency shift for PBASE in methanol. The larger frequency shift observed with methanol was attributed to the possible multilayer adsorption of PBASE rather than the formation of a well-defined monolayer, potentially due to solubility differences. Subsequent antibody immobilisation experiments (10 µg/mL Rabbit IgG Isotype Control) demonstrated that 2 mM PBASE in EtOH resulted in 1.44-fold higher antibody immobilisation. Based on the antibody mass change (Δm) measured by QCM-D, with values of 341.31 and 230.88 ng cm^-2^ for 2 mM PBASE in EtOH and methanol, respectively, the corresponding receptor densities were calculated to be 0.014 and 0.009 molecules nm^-2^ using Equations S1 and S3, respectively. Since our objective was to enhance antibody immobilisation, EtOH was selected as the optimal solvent for further studies. Next, PBASE concentration was investigated by comparing 1 mM and 2 mM PBASE in EtOH, as represented in Figures S4B and S4C, respectively. The QCM-D results showed that 2 mM PBASE yielded 2.31 times greater surface immobilisation than 1 mM PBASE. When antibody immobilisation was assessed under identical conditions (10 µg/mL Rabbit IgG Isotype Control), 2 mM PBASE led to a 1.07-fold increase in antibody binding compared to 1 mM. Although this difference was modest, the overall trend suggested that 2 mM PBASE in EtOH provided the most effective functionalisation, and it was used throughout the remainder of the study.


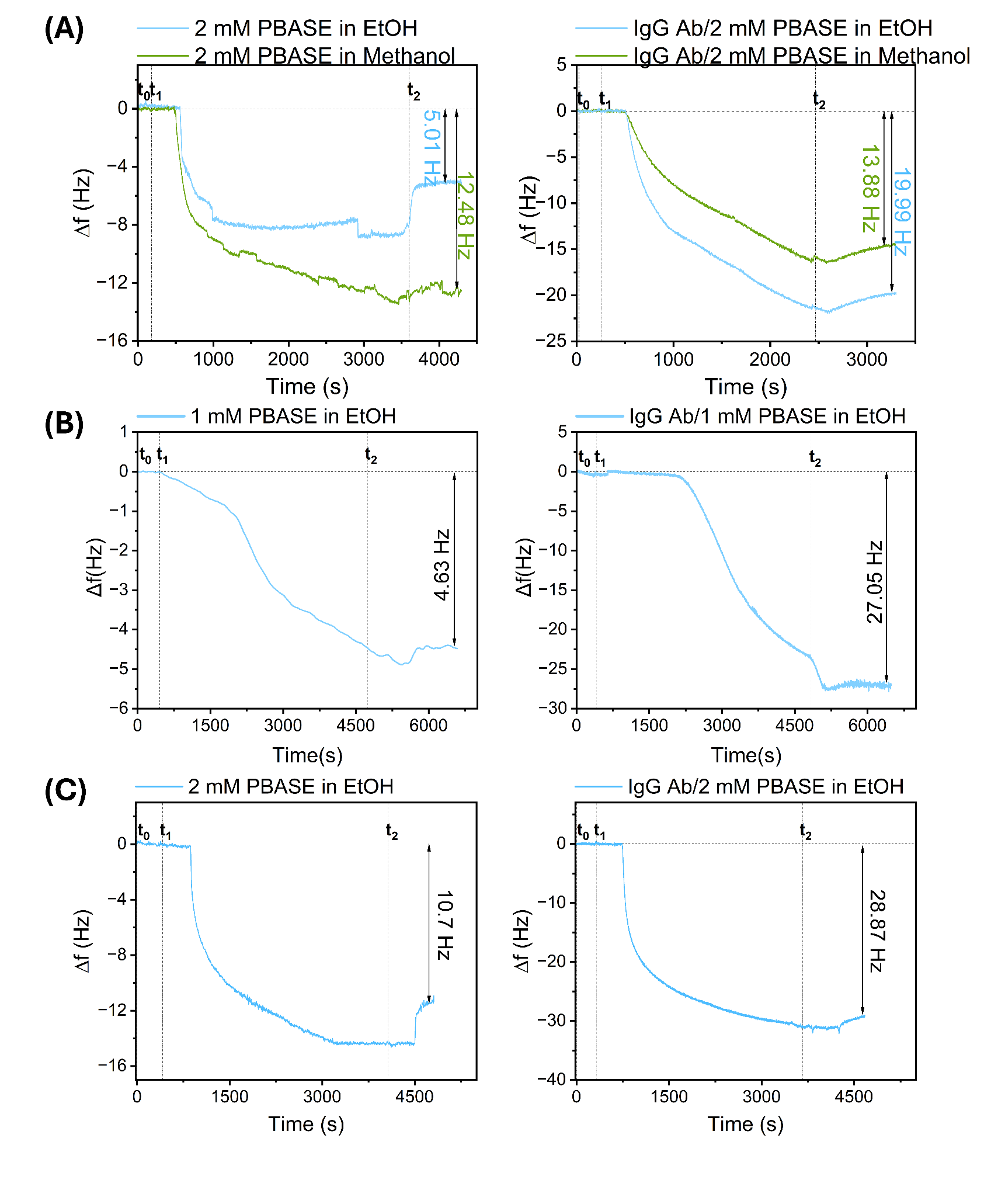


**Figure S4.** PBASE characterisation using QCM-D. (A) Frequency shifts obtained for the functionalisation of CVD-G with 2 mM PBASE in EtOH and 2 mM PBASE in methanol (left) and antibody immobilisation upon functionalisation (right). t_0_: EtOH/Methanol flow 40 µL/min t_1_: 2 mM PBASE in EtOH/Methanol flow 40 µL/min t_2_: EtOH/Methanol flow 40 µL/min. (B) Frequency shifts obtained for the functionalisation of CVD-G with 1 mM PBASE in EtOH (left) and antibody immobilisation upon functionalisation (right). t_0_: EtOH flow 40 µL/min. t_1_: 1 mM PBASE in EtOH flow 40 µL/min t_2_: EtOH flow 40 µL/min. (C) Frequency shifts obtained for the functionalisation of CVD-G with 2 mM PBASE in EtOH (left) and antibody immobilisation upon functionalisation (right). t_0_: EtOH flow 40 µL/min t_1_: 2 mM PBASE in EtOH flow 40 µL/min t_2_: EtOH flow 40 µL/min.

# **5. Details of Raman Measurement**

Table S1 presents the details of the three single-spectrum measurements, including the peak intensities for the D, G, and 2D bands, while Figure S5 presents full-range Raman spectra of pristine CVD-G and CVD-G/PBASE. The I_D_/I_G_ and I_2D_/I_G_ ratios were calculated based on these peak heights to assess structural disorder and doping effects.

**Table S1.** Three single-spectrum Raman measurements and their mean values for pristine CVD-G and CVD-G/PBASE.

| Peak heights | Pristine CVD-G | | | | CVD-G/PBASE | | | |
| --- | --- | --- | --- | --- | --- | --- | --- | --- |
|  | Y1 | Y2 | Y3 | Mean | Y1 | Y2 | Y3 | Mean |
| D | 233.955 | 184.276 | 177.640 | 198.623 | 3172.566 | 2619.476 | 3001.501 | 2931.181 |
| G | 2599.553 | 2987.818 | 2969.063 | 2852.145 | 9916.327 | 19886.562 | 16174.759 | 15325.883 |
| 2D | 5240.400 | 6229.121 | 6675.834 | 6048.452 | 34208.739 | 30273.510 | 34388.925 | 32957.058 |
| I_D_/I_G_ | 0.090 | 0.062 | 0.060 | 0.071 | 0.320 | 0.132 | 0.186 | 0.212 |
| I_2D_/I_G_ | 2.016 | 2.085 | 2.248 | 2.116 | 3.450 | 1.522 | 2.126 | 2.366 |

For a more statistically robust analysis, Raman mapping was performed over a 10 × 10 µm area, collecting 2116 spectra per sample. The mapping was conducted with a 515 nm excitation laser, with 1800 lines/mm grating, and 10% laser power to prevent sample damage. Each spectrum was acquired with an exposure time of 1 second, using three accumulations per point to improve the signal-to-noise ratio. The spatial resolution was set to 0.1 µm per step, ensuring the high-resolution characterisation of graphene uniformity and functionalisation effects. All collected spectra were processed and fitted using Renishaw software, with peak fitting performed for the D, G, and 2D peaks. This fitting ensured the accurate determination of peak positions and intensities, enabling reliable comparisons between pristine and functionalised graphene samples.


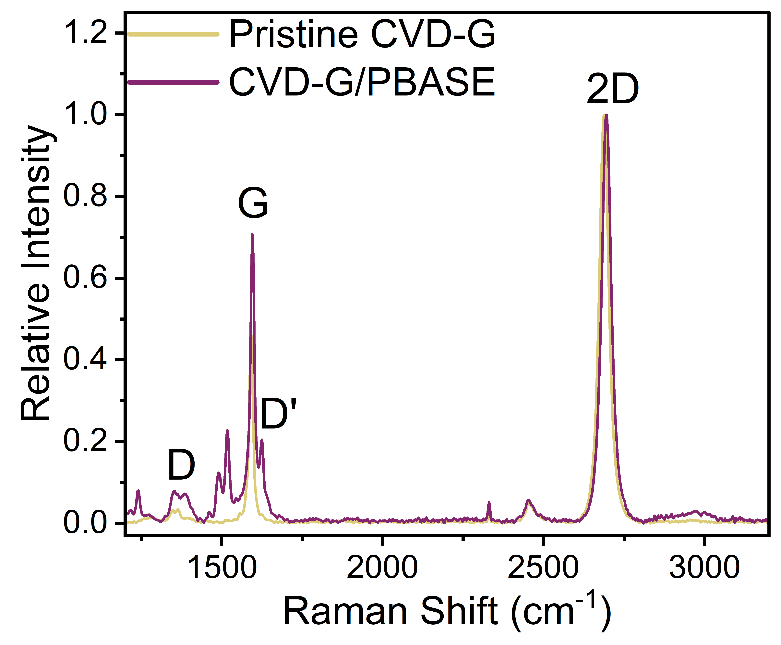


**Figure S5.** Full range Raman spectra of Pristine CVD-G and CVD-G/PBASE.

# **6. X-ray Photoelectron Spectroscopy (XPS) Survey Spectrum of CVD-G/PBASE**

The XPS spectra of CVD-G/PBASE exhibit characteristic peaks at C 1s (~284.8 eV), O 1s (~532 eV), and N 1s (~400 eV). This is consistent with the expected elemental composition, as shown in Figure S6. The N 1s peak confirms the presence of the NHS-terminated PBASE adlayer on graphene, consistent with effective functionalisation via *π-π* adsorption of the pyrene moiety. The measured binding energies for these core levels align well with literature values, further validating the chemical assignment of the CVD-G/PBASE.


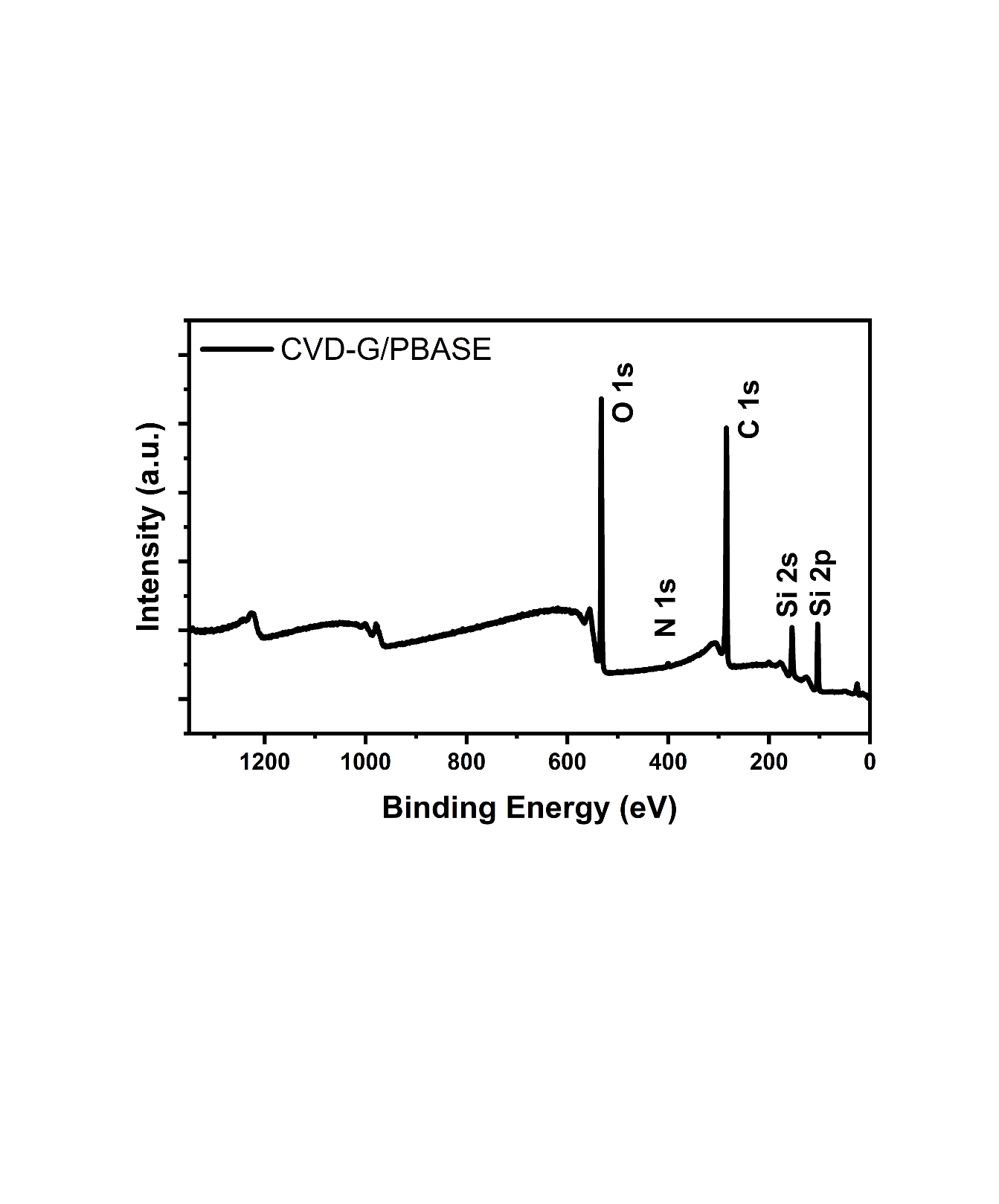


**Figure S6**. XPS survey spectra of CVD-G/PBASE.

**7. Quantification of PBASE Surface Density Using QCM-D (Sauerbrey Equation)**

To calculate the molecular density of PBASE from QCM-D measurements, the Sauerbrey equation was applied. The relationship between QCM resonance frequency shifts and mass changes was first described by Günter Sauerbrey in 1959 and formulated in the Sauerbrey equation.^[2]^ This equation establishes a linear correlation between the frequency shift (*Δf*) of an oscillating quartz crystal and the corresponding mass change (*Δm*), and is defined by Equation S1:

$\Delta m=-C\cdot\frac{\Delta f}{n}$ (S1)

where *C* is the sensitivity constant, and *n* is the harmonic number. For a 5 MHz crystal, *C* is 17.7 ng cm^-2^ Hz^-1^. The parameter *n* represents the odd harmonics (1, 3, 5, 7, …). In this study, the resonance frequency shift (*Δf*) was measured as 5 Hz at n = 7, corresponding to a surface mass change (Δm) of 89.38 ng cm^-2^, as determined using QSense Find software. Using this Δm value, further calculations were performed to determine the thickness of the PBASE functionalisation layer (t) based on Equation S2:

$t=\frac{\Delta m}{\rho}$ (S2)

where *ρ* is the solvent density. For EtOH (*ρ* = 785 kg m^-3^ at 25 °C), the functionalisation layer thickness was calculated to be 1.14 nm. Lastly, the molecular density (*N*) of the PBASE layer was determined by applying Equation S3:

$N=\frac{\Delta m}{{MW}_{PBASE}}N_{A}\times{10}^{-14}$ (S3)

where MW_PBASE_ is the molecular weight of PBASE (385.41 g mol^-1^), and N_A_ is Avogadro’s number (6.02 x 10^23^ molecules mol^-1^). The molecular density was found to be ~1.4 molecules nm^-2^. These calculations were repeated across all measurements to ensure accuracy and reproducibility.

**8. Quantification of PBASE Surface Density Using I-V Transfer Curves**

To determine the molecular density of PBASE using I-V transfer curves, the electric double-layer gated GFET was treated as a planar capacitor.^[3]^ The PBASE-induced charge on graphene was calculated using Equation S4:

$\text{σ}=\frac{\varepsilon}{\lambda_{D}}\times\text{Δ}V_{CNP}=C\times\frac{\text{Δ}\text{V}_{\text{CNP}}}{A}$ (S4)

where *C* is capacitance, *ΔV_CNP_* is the shift in the Dirac point after PBASE functionalisation on graphene (65 mV), *A* is the area of the semiconducting channel (8100 µm^2^), *ε = ε_PBS_ x ε_0_* (e_PBS_ = 78.7, and e_0_ = 8.854 x 10^-12^ F m^-1^). For 1x PBS at 25°C, the theoretical Debye length $\lambda_{D}$= 0.76 nm ^[4]^. Substituting the respective values gives an area charge density $\sigma$of 5.96 x 10^-20^ C nm^-2^, or, equivalently, 0.372 e nm^-2^. The PBASE density was then calculated by dividing this charge density by the charge per PBASE molecule known from the literature (~ 0.25e, e = 1.602 x 10^-19^ C),^[5]^ resulting in an estimated density of ~1.49 molecules nm^-2^. The I-V transfer curves used for this calculation are presented in Figure S7.

**Figure S7.** I-V transfer curves of pristine CVD-G and CVD-G/PBASE.


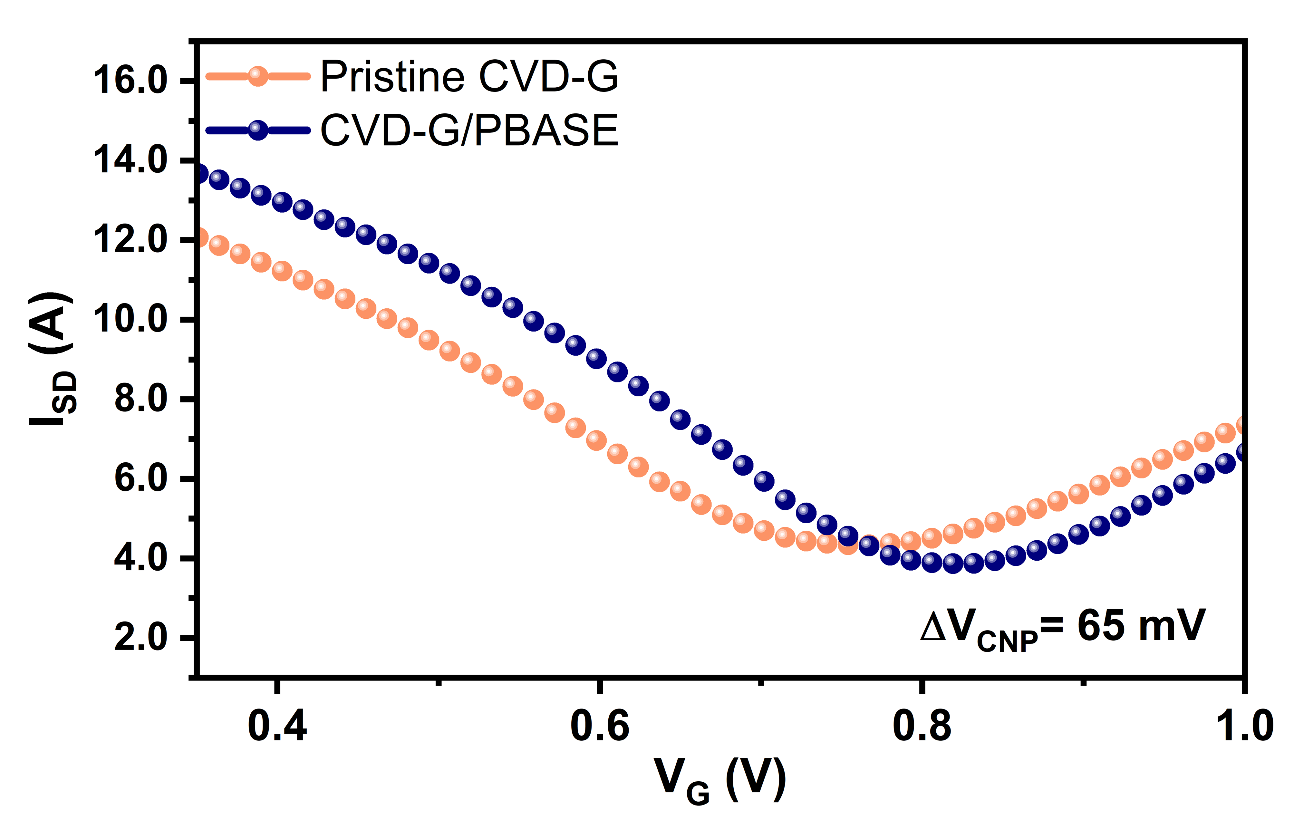


**9. Quantification of PBASE Surface Density Using XPS**

The surface density of PBASE adsorbed onto the graphene layer was determined by quantifying the atomic ratio of nitrogen as a unique marker of the PBASE molecule to the total carbon that was contributed by both the graphene substrate and the PBASE adlayer. XPS analysis was performed on two samples: bare graphene (for reference) and PBASE-functionalised graphene. High-resolution core-level scans for C 1s and N 1s were obtained. The raw peak areas were converted into atomic percentages (At. %) using the relative sensitivity factors of the instrument, where the C 1s signal was set to 1.00 and the N 1s signal to 1.80 (for the Al K-alpha source). The overall measured At. % values extracted were C ≈ 98.21 At.% and N = 1.78 At.%.

To calculate the surface density, we assumed the following. Firstly, the pristine graphene basal plane is assumed to have an atomic density (N_graphene_) of 3.8 X 10^15^ carbon atoms cm^-2^, or 38.2 atoms nm^-2^. Secondly, based on the molecular formula for 1-pyrenebutanoic acid N-hydroxysuccinimide ester (C_24_H_17_NO_4_), the number of nitrogen atoms per PBASE molecule (n_N_) = 1, and the number of carbon atoms per PBASE molecule (n_C_) = 24. Thirdly, the PBASE layer is assumed to be a uniform monolayer, and the XPS photoelectron escape depth is sufficiently large that the measured C 1s signal originates from both the surface PBASE layer and the underlying graphene substrate.

The measured atomic ratio of nitrogen to carbon (R) is given by the ratio of the total number of nitrogen atoms on the surface to the total number of carbon atoms (both from PBASE and graphene) per unit area using Equation S5:

R= (N/C)_meas_ = $\frac{\text{At.}\text{ }\text{\% N}}{\text{At.}\text{ }\text{\% C}}$ = $\frac{\text{1.78}}{\text{98.21}}=0.01812$ (S5)

The numerator (N signal) originates only from PBASE, while the denominator (C signal) originates from both PBASE and the underlying graphene. If (Γ) is the PBASE surface density in molecules nm^−2^, the total atomic counts are:

$$Total N atoms= \text{Γ}\text{.}n_{N}$$

$$Total C atoms= N_{graphene}\text{+ }\text{Γ}\text{.}n_{C}$$

This yields:

$$R=\frac{\text{Γ}n_{N}}{N_{graphene}+\text{Γ}n_{C}}$$

Subsequently,

$\text{Γ}\text{=}\frac{N_{graphene}}{n_{N}-{Rn}_{C}}$ = $\frac{0.01812 \times38.2}{1-0.01812\times24}=1.23 molecules {nm}^{-2}$

The surface density of PBASE on the graphene surface was calculated to be 1.23 molecules per nm^2^. This corresponds to an average surface area per molecule of A=1/Γ=0.82 nm^2^/molecule. The corresponding XPS N 1s and C 1s spectra are presented in Figure 2D.

# **10. Device Performance of IgG aNfL-immobilised GFETs**

The device performance of IgG aNfL-immobilised GFETs was evaluated in terms of their electrical response. The transfer curves in plasma obtained show a positive shift with increasing concentrations of neurofilament light (NfL), as shown in Figure S8A. The response comparison of PBS and plasma measurements is presented in Figure S8B. The response change between different matrices was found not to be statistically significant for different concentrations. The quantitative analysis of the results obtained and the standard curve is discussed in Section 2.3.

**Figure S8.** Electrical response of IgG aNfL immobilised GFET. (A) I-V transfer curves were obtained using five known concentrations of NfL in plasma. (C) Signal intensity comparison between PBS and plasma matrices revealed no significant difference (n=3, p = 0.12).


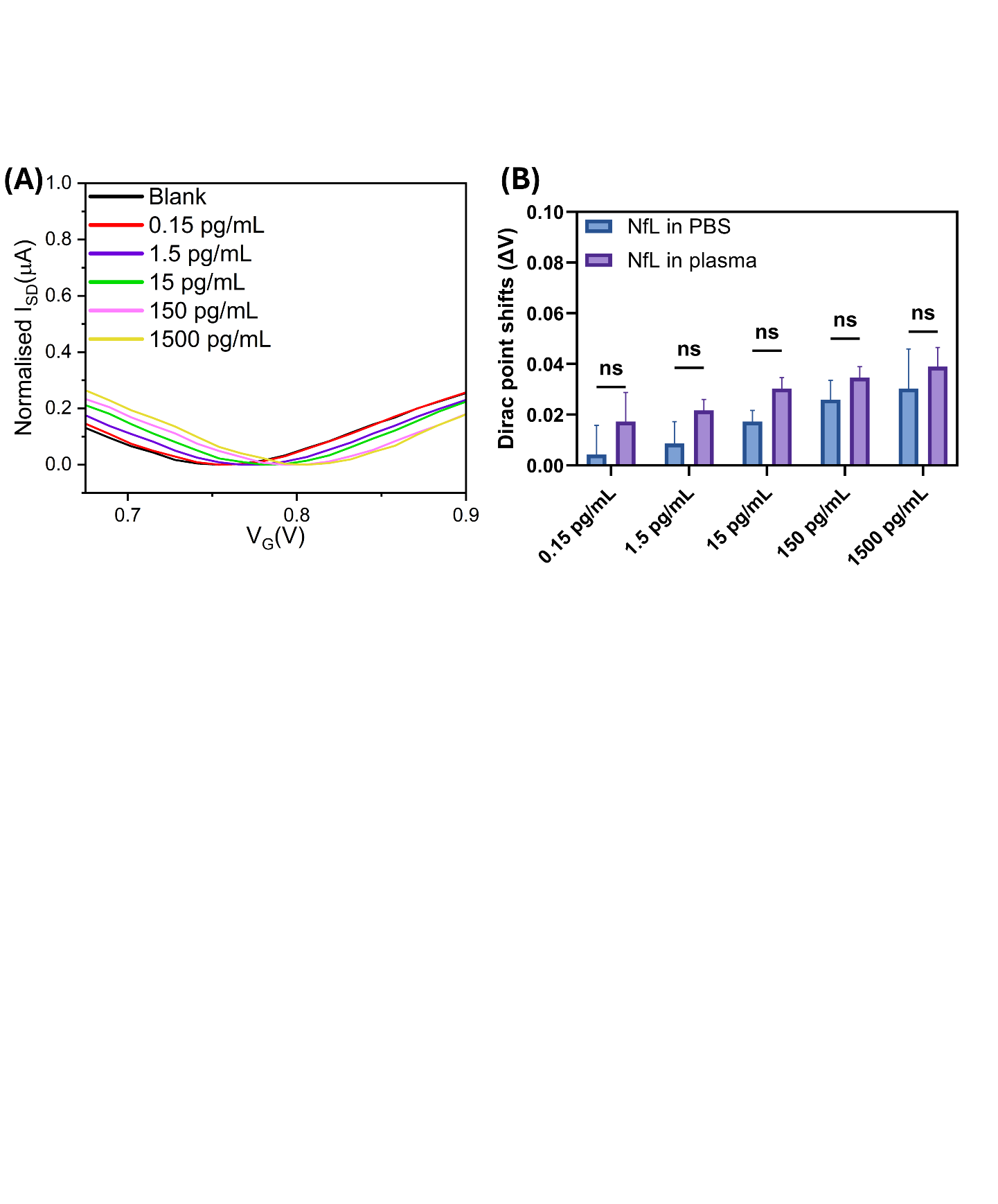


# **11. Analytical Performance of F(ab’)_2_ aNfL-immobilised GFETs**

Real-time measurements were conducted to assess the dynamic sensor response (Figure S9A). Upon switching from PBS to healthy plasma, the I_SD_ current was transiently perturbed before returning to baseline, confirming a minimal non-specific response to the plasma matrix. The subsequent addition of 0.15 pg/mL NfL produced a distinct and sustained decrease in I_SD_, demonstrating specific analyte detection. To evaluate selectivity, the cross-reactivity with structurally related neurodegeneration biomarkers (GFAP, Tau316, and pTau217) was assessed. F(ab)_2_ aNfL-functionalised GFETs showed a negligible response to all tested interferents, confirming high specificity for NfL (Figure S9B).


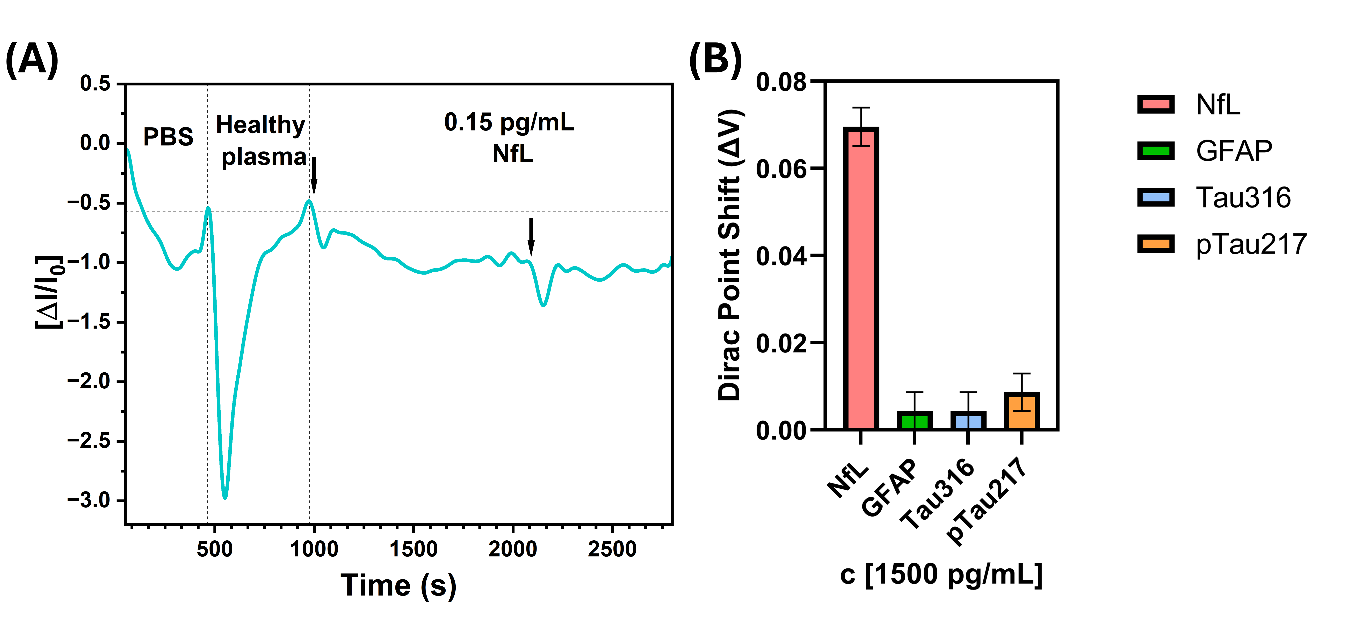


**Figure S9.** Analytical performance and stability characterisation of F(ab’)_2_ aNfL-functionalised GFETs. (A) Real-time response of the GFET device for the detection of NfL in plasma. Dash lines indicate the change of solutions. The arrows show the addition of NfL. (B) Selectivity assessment showing the Dirac point shift in response to NfL and structurally related neurodegeneration biomarkers (GFAP, Tau316, and pTau217) at a fixed concentration.

# **12. Quality Control of F(ab’)_2_ aNfL**

In order to assess the quality of the processed F(ab’)_2_ aNfL, a bicinchoninic acid (BCA) assay, sodium dodecyl sulphate-polyacrylamide gel electrophoresis (SDS-PAGE), and an indirect enzyme-linked immunosorbent assay (ELISA) were conducted sequentially. The BCA assay was used to determine the total protein concentration. This assay relies on the reduction of copper (II) sulphate by peptide bonds, leading to a colorimetric change from green to purple proportional to protein concentration, which is measured spectrophotometrically. The Pierce™ BCA Protein Assay Kit (Thermo Fisher) was used for this analysis. A standard curve was first generated using bovine serum albumin (BSA) as the standard protein, with serial dilutions of BSA prepared starting at 1000 µg/mL (1:2 dilution). Working reagents were prepared by combining 50 parts of reagent A with 1 part of reagent B, and 200 µL of this mixture was added per well. 10 µL of F(ab’)_2_ aNfL was used for analysis, with all samples run in triplicate. After incubation at 37 °C for 30 minutes, the plates were cooled to room temperature, and absorbance was measured at 562 nm. Linear regression analysis yielded an R^2^ value of 0.999 (Y = 0.0012 * X + 0.125), ensuring high accuracy. The interpolated concentration of F(ab’)_2_ aNfL was determined from three independent experiments, yielding values of 178.75, 185.45, and 207.15 µg/mL, resulting in an average concentration of 190.45 µg/mL. The standard curve and interpolated values are presented in Figure S10.


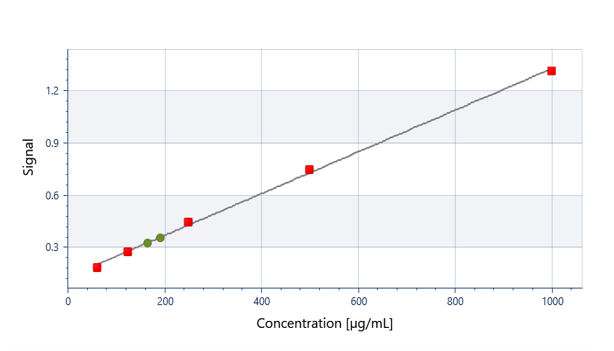


**Figure S10.** Standard curve obtained using BCA to determine the concentration of processed F(ab’)_2_ aNfL (R^2^= 0.999). The red squares show the average of known concentrations of BSA, and green circles show the duplicate of interpolated concentration of F(ab’)_2_ aNfL.

SDS-PAGE was performed to evaluate the integrity of the processed F(ab’)_2_ aNfL fragments. A total of 70 mL of TGS (Tris-Glycine-SDS) running buffer was prepared and diluted to 700 mL with DI water. Samples were prepared under reducing (with β-mercaptoethanol, BME) and non-reducing (Laemmli buffer only) conditions, each containing 1 µg of protein, 6 µL of buffer, and 0.5 µL of BME when applicable, followed by denaturation at 80°C for 10 minutes. The protean gel cassette was assembled, and samples were loaded into the wells before running at 180 V for 40 minutes. Following electrophoresis, the chamber and cassette were disassembled, and gel images were captured under UV excitation. For indirect ELISA, Nunc MaxiSorp ELISA plates were coated with 1 µg/mL NfL and incubated overnight at 4°C before washing with PBS. Blocking was performed using 2% BSA for one hour at 37°C, followed by decanting. Serial dilutions of IgG aNfL, F(ab’)_2_ aNfL, and control F(ab’)_2_ aNfL were applied, starting at 250 ng/mL (1:3 dilution), and incubated for one hour at 37°C. After three washes with PBS-Tween 20, HRP-conjugated anti-rabbit IgG (light chain specific) was added and incubated under the same conditions. Following another series of washes, TMB substrate was introduced, and the reaction was allowed to develop in the dark for ~5 minutes before being stopped with sulphuric acid. Absorbance was measured at 450 nm. These analyses confirmed the purity and antigen-binding functionality of the processed F(ab’)_2_ fragments, ensuring their suitability for further biosensing applications.

# **13. Confirmation of Antibody Immobilisation**

The acquired AFM topography of IgG aNfL and F(ab’)_2_ aNfL immobilised graphene yielded surface roughness (R_a_) values of 0.694 and 0.423 nm, respectively (Figure S11A). Three-dimensional AFM topography on graphene was obtained after sequential functionalisation with F(ab’)_2_ aNfL, BSA blocking, and subsequent NfL incubation. The average R_a_ values increased from 0.423 nm to 0.570 nm and 1.140 nm, respectively, indicating a monotonic rise in surface roughness with each step (Figure S11B). This trend is consistent with successive molecular layer formation and protein binding, which introduce additional nanoscale features and modest heterogeneity. To further support these findings, XPS analysis was employed. The XPS survey spectra of both CVD/PBASE/IgG aNfL and CVD/PBASE/F(ab’)_2_ aNfL are presented in Figure S11C. Both spectra include the essential core-level peaks (C 1s, O 1s, and N 1s), confirming the presence of antibody immobilisation. In addition, examination of the N 1s peak shows a consistent increase with each step of PBASE, antibody immobilisation and BSA blocking, corroborating the incremental addition of nitrogen-containing layers on the graphene surface. (Figure S11D).

**Figure S11.** Confirmation of antibody immobilisation. (A) The AFM topography results show the average roughness of the graphene surface after immobilisation with IgG aNfL and F(ab')_2_ aNfL. (B) 3D AFM topography of graphene surfaces after immobilisation with F(ab')_2_ aNfL, BSA, and bioconjugation with NfL protein revealed a monotonic rise in surface roughness. (C) XPS survey spectra of graphene surface after immobilisation with IgG aNfL (left) and F(ab')_2_ aNfL (right). Both spectra exhibit the same characteristic peaks: O 1s, N 1s, C 1s, Si 2s, and Si 2p. (D) XPS N 1s core spectra of graphene after functionalisation with PBASE, followed by immobilisation with IgG NfL and BSA blocking. The presence and consistent increase of the N 1s peak confirms the successful functionalisation of biomolecules onto the graphene surface.


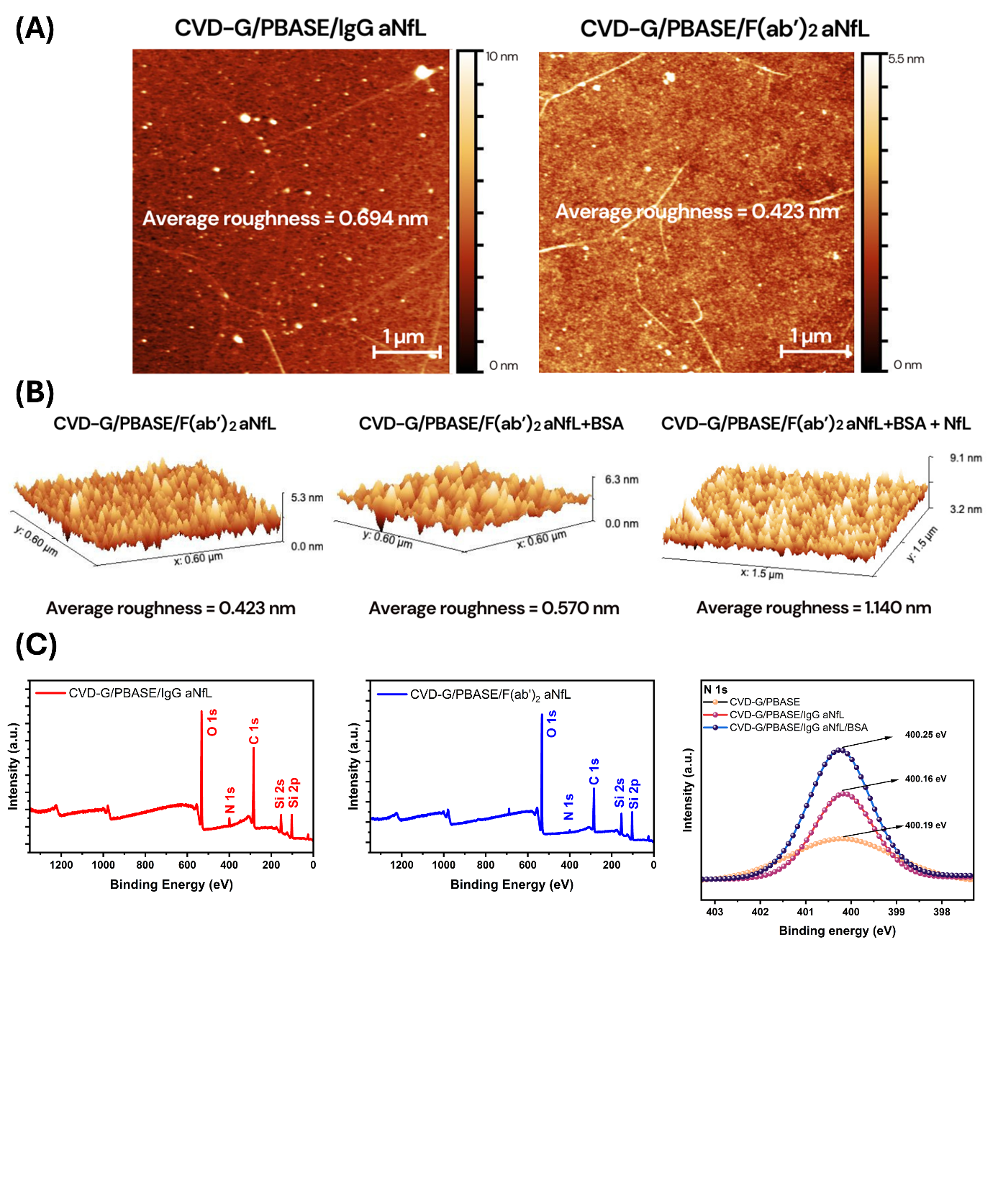


# **14. Quantification of Antibody Surface Density Using QCM-D**

To ensure an equivalent number of molecules across different antibody solutions, the required concentrations and volumes were calculated for two antibody types: F(ab’)_2_ aNfL (rabbit F(ab’)_2_, MW $\cong$ 88 kDa); and IgG aNfL (whole IgG, MW $\cong$ 150 kDa). Using a final concentration of 2 µg/mL for F(ab’)_2_ aNfL in a 1.52 mL solution, the total mass was determined to be 3.04 µg. The mass (m) was converted into moles (n) from the molecular weight (M_W_) using Equation S6:

$n= \frac{m}{M_{W}}$ (S6)

This mass corresponds to approximately 3.45 × 10^-11^ mol for F(ab’)_2_ aNfL. The number of molecules (N) is derived based on Avogadro’s number (N_A_ = 6.022×10^23^) using Equation S7:

$N=n \times N_{A}$ (S7)

The number of molecules corresponds to 2.08 × 10^13^ for F(ab’)_2_ aNfL. To maintain molecular equivalence, the required IgG aNfL concentration was determined to be 3.41 µg/mL, corresponding to a total mass of 5.18 µg. Both antibody solutions were standardised to 15 nM.

Although this concentration was not sufficient to achieve signal saturation, it prevented accurate thickness estimations via QCM-D. This limitation arises because QCM-D provides a concentration-dependent response.^[6]^ Despite this, the technique still allowed for the quantitative assessment of adsorption mass based on resonance frequency shifts (Δf) measured at the seventh overtone (n = 7) using QSense Find software and shown in Figure S12.

Following antibody immobilisation, the measured mass changes were 118 ng/cm² for IgG aNfL and 168 ng/cm² for F(ab’)_2_ aNfL, indicating a higher surface coverage for the F(ab’)_2_ fragments. Subsequent blocking with 2% BSA further increased the mass values to 323.5 ng/cm² and 358.5 ng/cm² for IgG aNfL and F(ab’)_2_ aNfL respectively, suggesting the effective passivation of non-specific binding sites. After introducing 1.85 nM NfL for an hour, the final immobilised masses reached 343 ng/cm^2^ for IgG aNfL and 392.5 ng/cm^2^ for F(ab’)_2_ aNfL, confirming successful analyte binding.

To estimate receptor and analyte densities, the measured mass changes were converted into molecular surface coverage using Equation S3. The receptor density increased from 0.004 molecules/nm^2^ for IgG aNfL to 0.011 molecules/nm^2^ for F(ab’)_2_ aNfL. Given the molecular weight of NfL (81 kDa), the analyte density was similarly estimated using measured mass changes of 19.5 and 34 ng/cm^2^, showing an increase from 0.0014 to 0.0025 molecules/nm² for IgG aNfL and F(ab’)_2_ aNfL respectively. This 1.78-fold enhancement in analyte binding can be attributed to the higher immobilisation density of F(ab’)_2_ fragments on the graphene surface, facilitated by their smaller size compared to whole IgG antibodies.

**Figure S12.** Mass quantification of F(ab’)_2_ aNfL and IgG aNfL immobilisation using QCM-D. Vertical dashed lines indicate the approximate timeframes for each flow change, while the numerical values represent the final measurements obtained before the next flow step. t_0_: PBS flow 40 µL/min t_1_: 15 nM antibody/PBS flow 10 µL/min t_2_: 2% BSA/PBS flow 40 µL/min. t_3_: 1.85 nM NfL/PBS flow 40 µL/min.


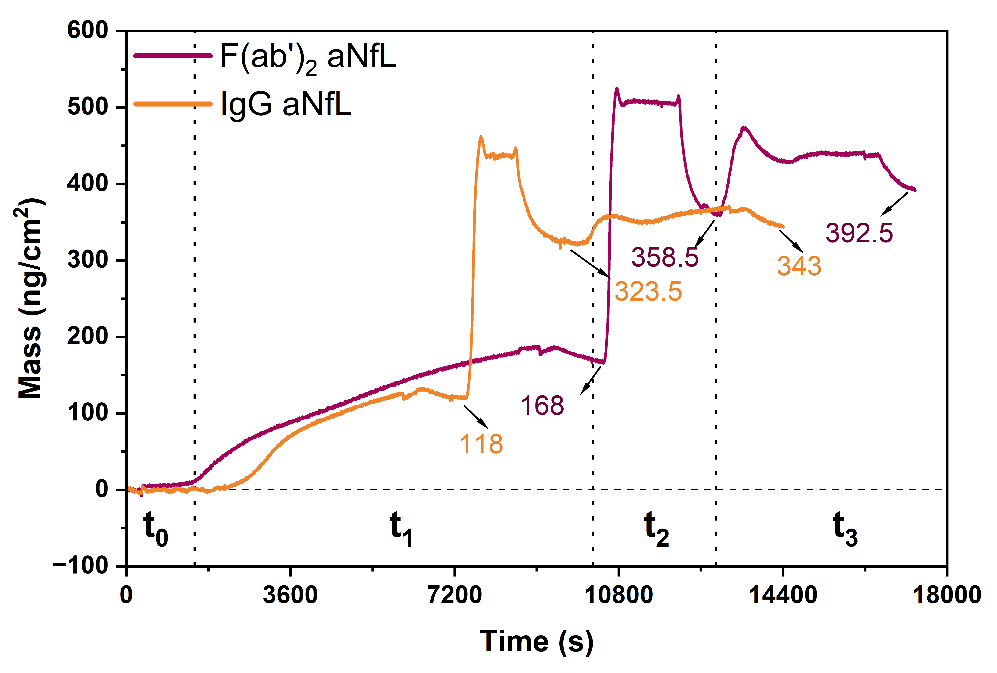

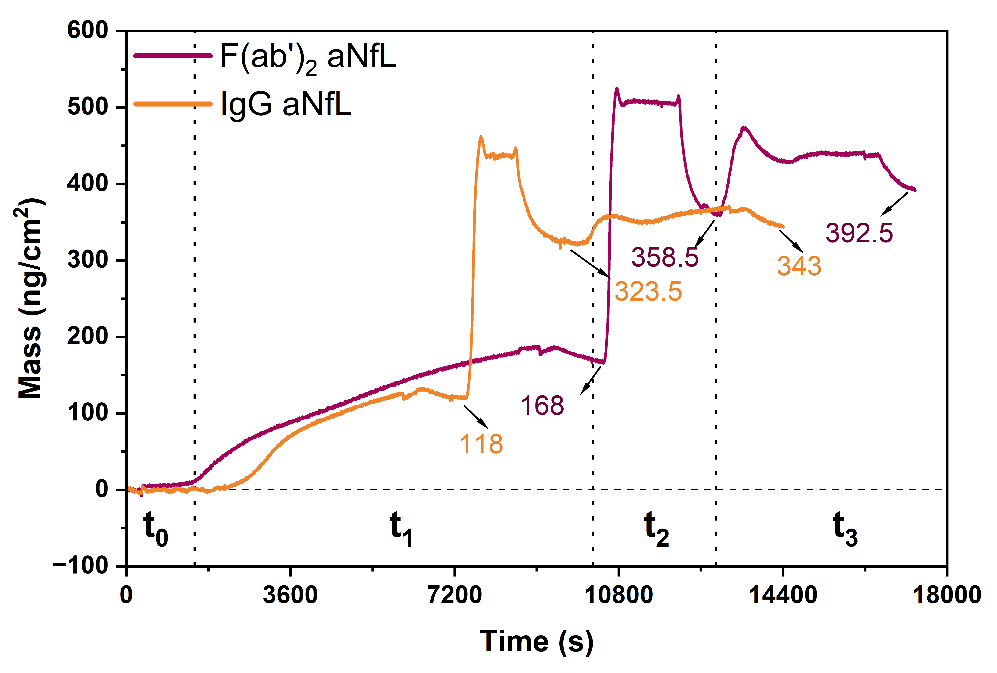


# **15. Quantification of Antibody Surface Density Using I-V Transfer Curves**

To determine the receptor density of antibodies from I-V transfer curves, the charge doping induced by NfL bioconjugation ($\sigma$) was estimated using the same approach as for PBASE density calculations using Equation S4:

$\sigma=\frac{\varepsilon}{\lambda_{D}}\times\Delta V_{CNP}=C\times\frac{{\Delta V}_{CNP}}{A}$ (S4)

where ΔV_CNP_ is the shift in the Dirac point after NfL binding on graphene (39 mV for IgG aNfL and 65 mV for F(ab’)_2_ aNfL). λ_D_ was calculated based on 0.01× plasma ionic strength (I = 0.0015 M) ^[7]^ using Equation 1, yielding a value of λ_D_​ of 7.85 nm. Substituting these values gives charge densities $\sigma$ of 3.46 x 10^-21^ C nm^-2^ (0.0216 e nm^-2^) for IgG aNfL and 5.77 x 10^-21^ C nm^-2^ (0.0360 e nm^-2^) for F(ab’)_2_ aNfL. To calculate the receptor molecular density, the reported charge densities per receptor of -10.6 e for IgG aNfL and -4.3 e for F(ab’)_2_ aNfL were used.^[8]^ Dividing the overall charge density by the charge per receptor yields receptor densities of 0.0020 molecules nm^-2^ for IgG aNfL and 0.0084 molecules nm^-2^ for F(ab’)_2_ aNfL. The I-V transfer curves used for these calculations are shown in Figure S13. It is important to note that, while the charge values employed were not specific to the antibodies used in this study, they were selected based on structural similarity and therefore provide a reasonable approximation for the estimation calculations.


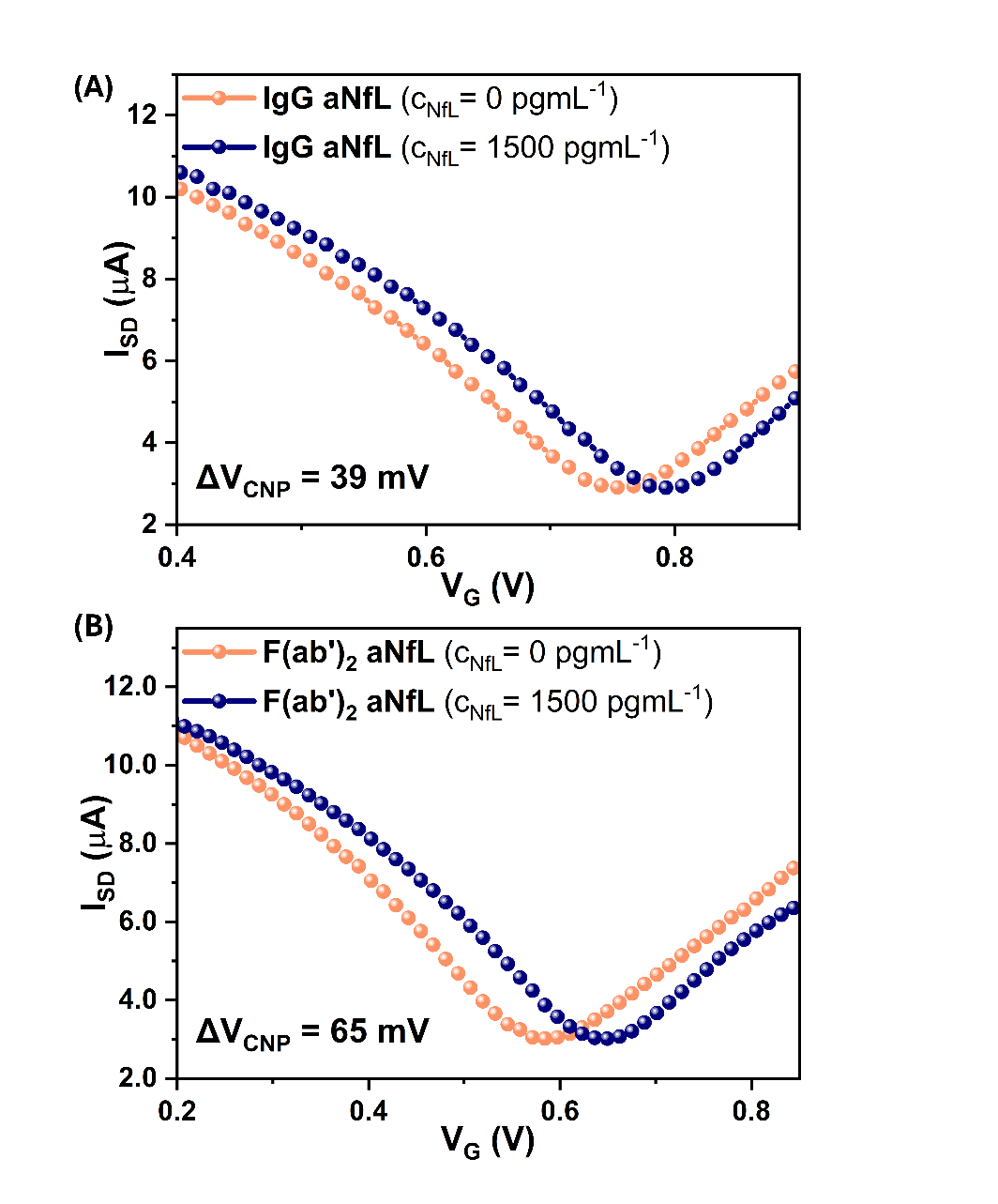


**Figure S13.** (A) I-V transfer curves showing the device response of IgG aNfL-immobilised at 0 pg mL^-1^ and 1500 pg mL^-1^ of NfL. (B) I-V transfer curves showing the device response of F(ab’)_2_ aNfL-immobilised at 0 pg mL^-1^ and 1500 pg mL^-1^ of NfL.

# **16. Clinical Sample Measurements with Single Molecule Array (Simoa) and GFET Platform**

Table S2 presents the interpolated NfL concentrations obtained from Simoa and GFET platforms for patient samples labelled P1 to P5. For each sample, the table reports the mean concentrations derived from replicate measurements, along with their corresponding coefficients of variation (CV%).

**Table S2.** Interpolated concentrations of NfL in five patient samples using Simoa and GFET

| Sample ID | Concentration by Simoa | | Concentration by GFET | |
| --- | --- | --- | --- | --- |
|  | Average of measured results | CV (%) | Average of measured results | CV (%) |
| P1 | 4.76 | 12.28 | 5.62 | 0.00 |
| P2 | 19.95 | 1.73 | 16.77 | 20.00 |
| P3 | 86.43 | 3.47 | 129.98 | 27.35 |
| P4 | 183.78 | 2.99 | 269.78 | 9.72 |
| P5 | 505.86 | 3.96 | 580.97 | 8.94 |

**References**

[1] G. Wu, X. Tang, M. Meyyappan, K. W. C. Lai, *Appl. Surf. Sci.* **2017**, *425*, 713.

[2] G. Sauerbrey, *Zeitschrift für Physik* **1959**, *155*, 206.

[3] A. Silvestri, J. Zayas-Arrabal, M. Vera-Hidalgo, D. Di Silvio, C. Wetzl, M. Martinez-Moro, A. Zurutuza, E. Torres, A. Centeno, A. Maestre, J. M. Gómez, M. Arrastua, M. Elicegui, N. Ontoso, M. Prato, I. Coluzza, A. Criado, *Nanoscale* **2023**, *15*, 1076.

[4] X. Lu, A. Miodek, W. M. Munief, P. Jolly, V. Pachauri, X. Chen, P. Estrela, S. Ingebrandt, *Biosens. Bioelectron.* **2019**, *130*, 352.

[5] W. Zhuang, H. J. Jang, X. Sui, B. Ryu, Y. Wang, H. Pu, J. Chen, *ACS Appl. Mater. Interfaces* **2024**, *16*, 27961.

[6] P. Hampitak, D. Melendrez, M. Iliut, M. Fresquet, N. Parsons, B. Spencer, T. A. Jowitt, A. Vijayaraghavan, *Carbon N.Y.* **2020**, *165*, 317.

[7] A. K. Covington, R. A. Robinson, *Anal. Chim. Acta.* **1975**, *78*, 219.

[8] D. Yang, R. Kroe-Barrett, S. Singh, T. Laue, *Antibodies* **2019**, *8*, 24.
